# Deflation-Corrected Estimators of Reliability

**DOI:** 10.3389/fpsyg.2021.748672

**Published:** 2022-01-04

**Authors:** Jari Metsämuuronen

**Affiliations:** Finnish National Education Evaluation Centre (FINEEC), Helsinki, Finland

**Keywords:** reliability, deflation in reliability, item-score correlation, deflation in correlation, coefficient alpha, coefficient theta, coefficient omega, maximal reliability

## Abstract

Underestimation of reliability is discussed from the viewpoint of deflation in estimates of reliability caused by artificial systematic technical or mechanical error in the estimates of correlation (MEC). Most traditional estimators of reliability embed product–moment correlation coefficient (PMC) in the form of item–score correlation (*Rit*) or principal component or factor loading (*λ_*i*_*). PMC is known to be severely affected by several sources of deflation such as the difficulty level of the item and discrepancy of the scales of the variables of interest and, hence, the estimates by *Rit* and *λ_*i*_* are always deflated in the settings related to estimating reliability. As a short-cut to deflation-corrected estimators of reliability, this article suggests a procedure where *Rit* and *λ_*i*_* in the estimators of reliability are replaced by alternative estimators of correlation that are less deflated. These estimators are called deflation-corrected estimators of reliability (DCER). Several families of DCERs are proposed and their behavior is studied by using polychoric correlation coefficient, Goodman–Kruskal gamma, and Somers delta as examples of MEC-corrected coefficients of correlation.

## Introduction: Attenuation and Deflation in the Estimates of Reliability

Reliability of test score (*REL*) is used in several ways of which quantifying the amount of random error in a score variable generated by a compilation of multiple test items may be the most concrete one in the measurement modeling settings. The formula of the average standard error of the measurement $S.E.m.=\sigma_{E}=\sigma_{X}\sqrt{1-R\,E\,L}$ is derived strictly from the basic definition of reliability $R\,E\,L=\sigma_{T}^{2}\,/\,\sigma_{X}^{2}=1-\sigma_{E}^{2}\,/\,\sigma_{X}^{2}$, where $\sigma_{X}^{2}$, $\sigma_{T}^{2}$, and $\sigma_{E}^{2}$ refer to the variances of the observed score variable (*X*) and the unobserved true score (*T*) and error (*E*) related to the classic relation of *X* = *T* + *E* (Gulliksen, 1950). Reliability is also used in assessing the (overall) quality of the measurement, in correcting the attenuation of the estimates of regression or path models, in correcting the attenuation in correlations in validity studies and meta-analyses, and for providing confidence intervals around these estimates (see, e.g., Gulliksen, 1950; Schmidt and Hunter, 2015; Revelle and Condon, 2018; Aquirre-Urreta et al., 2019). In all cases, the interest related to the accuracy of the estimates of reliability is understandable.

A less discussed challenge in the estimates by the traditional estimators of reliability is that their estimates may be radically *deflated* caused by artificial systematic errors during the estimation or *attenuated* as a natural consequence of random errors in the measurement (see the discussion of the terms in, e.g., Chan, 2008; Lavrakas, 2008; Gadermann et al., 2012; Revelle and Condon, 2018); deflation and its correction are the foci in this article. Empirical examples discussed later show that, in certain types of datasets, typically with very easy and very difficult tests and tests with incremental difficulty level including both easy and difficult items, the estimates of reliability may be deflated by 0.40–0.60 units of reliability (see, e.g., Zumbo et al., 2007; Gadermann et al., 2012; Metsämuuronen and Ukkola, 2019; see section “Practical Consequences of Mechanical Error in the Estimates of Correlation in Reliability”).

Guttman (1945) was the first to show the technical or mechanical underestimation in the estimators of reliability. He showed that all estimators in his family of estimators λ_1_ to λ_6_ underestimate the true population reliability. This result generalizes to such known estimators of reliability as Brown–Spearman prophecy formula (*ρbs*; Brown, 1910; Spearman, 1910), Flanagan–Rulon prophecy formula (*ρ_*FR*_*; Rulon, 1939), coefficient alpha (*ρ_α_*) generalized from Kuder and Richardson (1937) formula KR20 (ρ_*KR20*_) by Jackson and Ferguson (1941) and later named by Cronbach (1951), and estimators called the greatest lower bound (*ρ_*GLB*_*; e.g., Jackson and Agunwamba, 1977; Woodhouse and Jackson, 1977) because these are all special cases of λ_1_−λ_6_. Hence, using these estimators, the true (population) reliability is always underestimated. Later, Novick and Lewis (1967) pointed out that the underestimation related to the measurement modeling holds if the true values (taus) are not essentially identical and the error components related to the test items do not correlate (see the discussion also in Raykov, 2012; Raykov and Marcoulides, 2017).

Since Guttman (1945), the underestimation in *ρ_α_* has been handled in numerous studies and it has been connected to, among others, a simplified assumption of the classical test theory including unidimensionality, violations in tau–equivalence and latent normality, and uncorrelated errors (see discussion in, e.g., Green and Yang, 2009, 2015; Trizano-Hermosilla and Alvarado, 2016). Some scholars have been ready even to reject *ρ_α_* for all (see, e.g., Yang and Green, 2011; Dunn et al., 2013; Trizano-Hermosilla and Alvarado, 2016; McNeish, 2017) but the discussion is still going on. In many practical testing settings, even though better options are available, *ρ_α_* may still be used as one of the lower bound estimators of reliability because the basic assumptions of alpha such as unidimensionality and uncorrelated errors are usually met (e.g., Metsämuuronen, 2017; Raykov and Marcoulides, 2017).

On the top of attenuation related to the measurement modeling, the estimates of reliability are also deflated—sometimes radically as discussed above. The root cause for the deflation is that the estimates by product-moment correlation coefficient (PMC; Pearson, 1896) embedded in the traditional estimators of reliability in the form of item–score correlation (*Rit*) or principal- or factor loading (*λ_*i*_*) may be seriously deflated approximating 100% with items with extreme difficulty level and large sample size (see Metsämuuronen, 2020b,2021b). Deflation in PMC is caused by a phenomenon called here artificial systematic technical or mechanical error in the estimates of correlation (MEC). This phenomenon and its consequences are discussed in section “Mechanical Error in the Estimates of Correlation in PMC and some consequences.”

Replacing PMC in the estimators of reliability by a less-MEC-defected coefficient of correlation called later MEC-corrected estimators of correlation leads us to new kinds of estimators of reliability named here *deflation-corrected estimators of reliability* (DCER). DCERs can be divided into two types. One, focused on this article, are MEC-corrected estimators of reliability where PMC is replaced by a totally *different estimator* of correlation that is less prone to deflation than PMC. The other types of DCERs not discussed in this article could be called attenuation-corrected estimators of reliability; in these, PMC is replaced by relevant *attenuation-corrected estimators* of correlation. Some options for the latter are proposed by Metsämuuronen (2021c); attenuation corrected PMC and *eta*. The idea of DCER have been discussed (although not by this name) also, for instance, by Zumbo et al. (2007) and Gadermann et al. (2012) related to their ordinal alpha and ordinal theta; ordinal alpha and theta uses the matrix of inter-item *RPC*s instead of PMCs in the calculations and those are special cases of DCERs.

**FIGURE 1:**
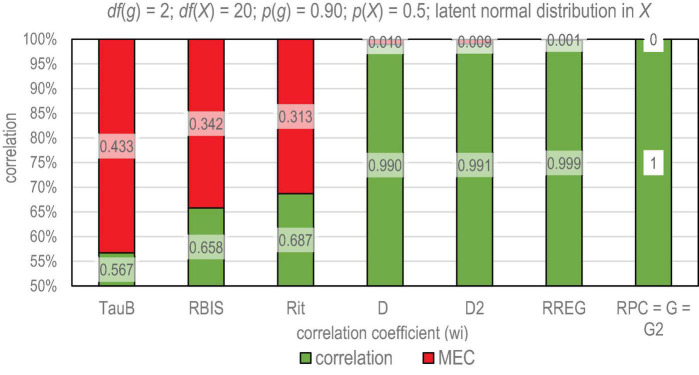
Magnitude of deflation in different estimators. TauB, Kendall tau-b; Rit, PMC; RBIS, biserial correlation; D, Somers delta (X dependent); D2, dimension-corrected D; RREG, r-polyreg correlation; RPC, polychoric correlation; G, Goodman-Kruskal gamma; G2, dimension-corrected G.

The crucial role of item–total correlation in the deflation of reliability has been discussed during the years (e.g., Metsämuuronen, 2009, 2016, 2017)^1^ and some options of corrected estimators of reliability have been initially suggested, however, without further studies of their behavior (see, e.g., Metsämuuronen and Ukkola, 2019; Metsämuuronen, 2020a,b, 2021b). According to simulations (see, e.g., Metsämuuronen, 2020b,2021b,2021d), some good alternatives for PMC are polychoric correlation coefficient (*RPC*; Pearson, 1900, 1913), Goodman–Kruskal gamma (*G*; Goodman and Kruskal, 1954), Somers delta (*D*; Somers, 1962), dimension-corrected *G* and *D* (*G*_2_ and *D*_2_; Metsämuuronen, 2020a,2021b) and bi- and polyreg correlation (see Livingston and Dorans, 2004; Moses, 2017). Notably, first, some estimators of item–score correlation may be found equally good alternatives or even better than *RPC*, *G*, or *D*. Second, although it seems that nonparametric coefficients of correlation based on order of the cases would be the best options for PMC, this is not categorically true. Of nonparametric options, Kendall’s tau-a (Kendall, 1938) and tau-b (Kendall, 1948), as examples, tend to underestimate true correlation even more than PMC (see Kendall, 1949; Metsämuuronen, 2021d; see Figure 1).

This article discusses the mechanisms of how the deflation related to coefficients of correlation causes deflation in the estimates of reliability and proposes several concrete options to solve the problem. Numerical examples are given of their behavior. It is asked, what is the effect of changing an estimator with a high quantity of deflation with an estimator with remarkably less deflation in the estimates of reliability? Section “Mechanical Error in the Estimates of Correlation in Product–Moment Correlation Coefficient and Some Consequences” discusses PMC as the root cause of the deflation in reliability, section “Deflation-Corrected Estimators of Reliability” discusses the conceptual base of the DCERs, and sections “Materials and Methods” and “Results” give numerical examples of how the deflation in the estimates of reliability is reduced when using DCERs instead of the traditional estimators.

## Mechanical Error in the Estimates of Correlation in Product–Moment Correlation Coefficient and Some Consequences

In measurement modeling settings, MEC refers to a characteristic of estimators of correlation to underestimate the true correlation between the test items (*g*_*i*_) and the latent trait θ manifested as a score variable (*X*) caused by artificial technical or mechanical reasons. In what follows, section “Product–Moment Correlation Coefficient, Mechanical Error in the Estimates of Correlation, and Deflation” discusses the overall effect of MEC in PMC, section “Sources of Mechanical Error in the Estimates of Correlation Affecting Deflation in Product–Moment Correlation Coefficient” discusses sources of MEC affecting deflation, section “Product–Moment Correlation Coefficient and the Estimators of Reliability” discusses how PMC is embedded in the estimators of reliability, and section “Practical Consequences of Mechanical Error in the Estimates of Correlation in Reliability” discusses what the effect of deflation in PMC in the estimates of reliability in the empirical dataset may be.

### Product–Moment Correlation Coefficient, Mechanical Error in the Estimates of Correlation, and Deflation

The phenomenon of attenuation in the estimates by PMC is well-known. Pearson (1903) and Spearman (1904) may be the first scholars discussing the mechanical errors in estimators of correlation, while Brown (1910) and Spearman (1910) may be the first to connect this to reliability. All of them tried to find a solution to the known challenge in the estimates of correlation known today as restriction of range (see the literature in Sackett and Yang, 2000; Sackett et al., 2007;, Meade 2010). It is known that when only a portion of the range of values of the variable is actualized in a sample it leads to inaccuracy in the estimates of PMC, that is, the values are attenuated. Schmidt and Hunter (1999), specifically, discusses the need of utilizing the knowledge from attenuation correction when estimating measurement error.

Even if there was no obvious restriction of range obtained due to a reduced variance in the score variable within the sample, PMC underestimates the true correlation always if the scales of the variables are not equal (see algebraic reasons in, e.g., Metsämuuronen, 2017). This kind of deflation in PMC caused by mechanical reasons is easy to illustrate by two identical continuous variables with an obvious perfect correlation, ρ_*XX*_ = 1. If we dichotomize one to be a binary variable (item *g*) and polytomize the other to include several ordinal or interval-scaled bins (score *X*), PCM between these variables cannot reach the obvious true (perfect latent) correlation. Instead, the value depends, among others, on the cut-off where the ordered continuous variable is dichotomized to 0s and 1s, that is, of the item difficulty. If the cut-off is extreme, PMC approximates 0 irrespective of the fact that the true correlation between the variables was perfect (see simulation e.g., in Metsämuuronen, 2021b). Even at the highest, PMC cannot reach the perfect ρ_*XX*_ = 1; if there are no ties in the score, the highest value approximates 0.866.^2^ Then, because of deflation, the loss of information in PMC may vary 13–100% depending on the item difficulty and the sample size. This loss of information is illustrated in Figure 1.

To give a practical illustration of the magnitude of error caused by deflation of correlation by different estimators, let us consider the situation described above: two identical variables with (obvious) perfect correlation ρ_*XX*_ = 1. Let there be 1000 cases and a normal distribution in the original variables. One of the variables becomes an item *g* by categorizing it into three categories (0, 1, and 2; *df*(*g*) = 2) and the other is polytomized into 21 categories (score *X, df*(*X*) = 20). The cut points are arbitrary from the illustration viewpoint; let the average difficulty level of the item be *p*(*g*) = 0.90 (or, *p*(*g*) = 0.10) that is, we have a very easy (or difficult) item, and the test score be of a medium difficulty level, *p*(*X*) = 0.50. Figure 1 illustrates the differences between some known estimators of correlation; the estimators are discussed later with literature.

Knowing that the latent correlation is perfect, the magnitude of the correlation strictly indicates the amount of deflation. We note that, of the estimators in the example, *tau-b*, biserial correlation (Pearson, 1909), and PMC (*Rit*) cannot reach the (obvious) perfect correlation between the two versions of the same variable and, more, the magnitude of deflation is remarkable (0.43, 0.34, and 0.31 units of correlation, respectively). Of the estimators, *D*, *D*_2_, and *RREG* give far better approximations of the latent correlation even if there still is some error in the estimates (0.010, 0.009, and 0.001 units of correlation, respectively). In contrast, *RPC*, *G*, and *G*_2_ reach the perfect latent correlation, that is, there is *no* deflation in the estimates when it comes to *difficulty level* of the items. Notably though, there may be other factors causing deflation or underestimation of association. Some of these factors are discussed in what follows (see also Metsämuuronen, 2021d).

### Sources of Mechanical Error in the Estimates of Correlation Affecting Deflation in Product–Moment Correlation Coefficient

By modifying the above example of two identical variables with relevant traditional coefficients of correlations such as *RPC*, *G*, and *D*, Metsämuuronen (2021b) concluded that PMC is affected (at least) by six sources of MEC: (1) *Discrepancy in scales of the variables in general*: PMC cannot reach the true (perfect) correlation between the item and the score when the dimensions of the variables differ from each other; (2) *Item difficulty and item variance*: the more extreme the item difficulty, the less variance, and the more underestimation in PMC. The loss of information approximates 100% with extremely easy and difficult items; (3) *The number of categories in the item*: the fewer the categories, the more underestimation in PMC; (4) *The number of categories in the score*: the fewer the categories, the lesser predictable the underestimation is; (5) *The number of tied cases in the score*: more there are tied cases in the score, lesser predictable the underestimation is. This is related to the sample size and the number of categories in the score (point 4); (6) *The distribution of the latent variable*: PMC underestimates the true correlation more if the latent variable is normal or skewed than in the cases of even distribution. These sources of the MEC are not the only possible ones although they are characteristics to PMC (see Metsämuuronen, 2021b).

Although rigorous studies have been done on these elements (e.g., Martin, 1973, 1978; Olsson, 1980; Anselmi et al., 2019; Metsämuuronen, 2021b) these tend to be fragmentary; systematic studies of the several elements of MEC would enrich our knowledge of the phenomenon. Notably, in all the six conditions above related to the attenuation in PMC, such benchmarking coefficients as *RPC* and *G* appeared to be MEC-free in the simulation (see Metsämuuronen, 2021b); the estimates reach the perfect correlation either strictly (*G* = 1) or asymptotically (*RPC*≈ 1) irrespective of the condition. *D* appeared to be less affected by MEC than PMC but not to the extent as *RPC* and *G* (see also Figure 1). The reason for the latter is that while *RPC* and *G* are not affected by the tied cases, *D* is, specifically, with short tests (see the differences of *D* and *G* also in Metsämuuronen, 2021a).

### Product–Moment Correlation Coefficient and the Estimators of Reliability

PMC is deep-rooted to the practices within the test theory and measurement modeling settings. From the reliability viewpoint, on the one hand, PMC is *strictly visible* in such classic estimators as ρ_*BS*_, ρ_*FR*_, ρ_*KR21*_, *ρ_α_*, *ρ_*GLB*_*, and λ_1_−λ_6_ discussed above. Common to these estimators is that the variance of the test score ($\sigma_{X}^{2}$) inherited from the basic definition of reliability is visible in the formula^3^ and $\sigma_{X}^{2}$, on its behalf, can be expressed by using the item–score correlation (*Rit* = *ρ*_*iX*_ = PMC): $\sigma_{X}^{2}={\left(\sum_{i=1}^{k}\sigma_{i}\times \rho_{i\,X}\right)}^{2}$(Lord et al., 1968) where *k* refers to number of items in the compilation and σ_*i*_to the standard deviations of partitions or items. Then, as an example, coefficient alpha can be expressed as (Lord et al., 1968):


(1) $$\rho_{\alpha }=\frac{k}{k-1}\,\left(1-\frac{\sum_{i=1}^{k}\sigma_{i}^{2}}{\sigma_{X}^{2}}\right)=\frac{k}{k-1}\,\left(1-\frac{\sum_{i=1}^{k}\sigma_{i}^{2}}{{\left(\sum_{i=1}^{k}\sigma_{i}\times \rho_{i\,X}\right)}^{2}}\right)$$

On the other hand, PMC is *embedded* in the estimators based on factor- and principal component analysis because the factor- and principal component loadings (λ_*i*_) are, essentially, correlations between an item and the score variable (e.g., Cramer and Howitt, 2004; Yang, 2010). This concerns such estimators of reliability as coefficient theta (*ρ*_*TH*_; Armor, 1973; see also Lord, 1958; Kaiser and Caffrey, 1965), known also as Armor’s theta:


(2) $$\rho_{T\,H}=\frac{k}{k-1}\,\left(1-\frac{1}{\sum_{i=1}^{k}\lambda_{i}^{2}}\right),$$

where *λ_*i*_* are principal component loadings of the (first or only) principal component, coefficient omega (*ρ*_ω_; Heise and Bohrnstedt, 1970; McDonald, 1970), known also as McDonald’s omega total:


(3) $$\rho_{\omega }=\frac{{\left(\sum_{i=1}^{k}\lambda_{i}\right)}^{2}}{{\left(\sum_{i=1}^{k}\lambda_{i}\right)}^{2}+\sum_{i=1}^{k}\left(1-\lambda_{i}^{2}\right)},$$

and coefficient rho, known also as maximal reliability (*ρ*_*MAX*_) or Raykov’s rho (Raykov, 1997a,^2004^) based on the conceptualization suggested by Li et al. (1996) and Li (1997):


(4) $$\rho_{M\,A\,X}=\frac{1}{1+\frac{1}{\sum_{i=1}^{k}\left(\lambda_{i}^{2}\,/\,\left(1-\lambda_{i}^{2}\right)\right)}}$$

(e.g., Cheng et al., 2012) where *λ_*i*_* are factor loadings.

From the traditional measurement modeling viewpoint (see, e.g., McDonald, 1999; Revelle and Condon, 2018) the forms in Eqs. (1) to (4) implicitly assume that *ρ*_*iX*_and λ_*i*_ are deflation-free. However, on the one hand, *ρ*_*iX*_ is known to be severely deflated (see above). On the other hand, if we use the operationalization familiar in principal component analysis (PCA), exploratory factor analysis (EFA), and structural equation modeling (SEM) where λ_*i*_ is a principal component- or factor loading, assumption of deflation-free estimates is too optimistic assumption because λ_*i*_ is, essentially, a correlation between item and the factor (or principal component) score variable (Yang, 2010). That is, λ_*i*_ is (essentially) *ρ*_*iX*_ being deflated as discussed above.

### Practical Consequences of Mechanical Error in the Estimates of Correlation in Reliability

The effect of MEC in deflation in the estimates of reliability may be remarkable. Two empirical examples are given. The first example comes from Gadermann et al. (2012) who report a dataset where, by using ordinal alpha (α_*ORD*_; Zumbo et al., 2007), another kind of DCER based on replacing the inter-item matrix of PMCs by a matrix of *RPCs*, the estimate by *ρ_α_* was deflated from 0.85 (α_*ORD*_) to 0.46 (*ρ*_α_), that is, 0.39 units of reliability which equals 46% (=0.85–0.46)/0.85).

Another example comes from a national level testing program of learning outcomes (*n* = 7,770; Metsämuuronen and Ukkola, 2019) where the preconditions of understanding the instruction language were assessed with a very easy 8-item, 11-point test. It was expected that only students with second language background in the instruction language would make mistakes in the test; of all test takers, 72% gave the full marks. The magnitude of the estimate of reliability by the traditional coefficient alpha was found to be *ρ_α_* = 0.25 and by rho ρ_*MAX*_ = 0.48. By using a DCER based on Somers *D* where *ρ*_*iX*_is replaced by *D*(*i*|*X*) = *D*_*iX*_ in the formula of alpha (see later Eq. 23), the magnitude of deflation-corrected alpha was *ρ_α_*DiX*_* = 0.86. Then, the magnitude of the estimate by *ρ_α_* was deflated around 0.60 units of reliability (71%) and the estimate by *ρ*_*MAX*_ around 0.38 units of reliability (44%). The obvious reason for the remarkably higher estimate by *ρ_α_*DiX*_* is that, in the case of binary items with extreme difficulty level, PMC as well as the factor loadings are severely attenuated while, in the binary case, *D* is less deflated. In both examples, the deflation in the estimates by the traditional estimators is remarkable. The latter example will be re-analyzed in section “Practical Example of Calculating Deflation-Corrected Estimators of Correlations Discussed in This Article” in details.

## Deflation-Corrected Estimators of Reliability

### Conceptual Base of the Deflation-Corrected Estimators of Reliability

Suggesting a radically new way of estimating reliability urges in-depth discussion of theoretical foundations of the new approach. However, here, the new concepts are built based on the traditional measurement models (see, e.g., McDonald, 1999; Cheng et al., 2012) which are, however, rethought and reconceptualized to also include the elements of deflation. Some further alternatives to consider for rethinking reliability are discussed in section “Options for Correcting the Deflation in Estimators of Reliability.” The effect of deflation is discussed here theoretically only to the extent that makes the notation in deflation-corrected estimators of reliability understandable.

Let *w*_*i*_ be a general weight factor that links the observed values (*x*_*i*_) of an item *g*_*i*_ with the latent variable θ manifested as a score variable:


(5) $$x_{i}=w_{\text{i}}\,\theta +e_{i}$$

generalized from the traditional one-latent variable model (e.g., McDonald, 1999; Cheng et al., 2012). It is relevant to assume that the weight factor *w*_*i*_ is a coefficient of correlation (−1≤*w*_*i*_≤ + 1) such as *Rit*, *RPC*, *G*, or *D*, or principal component- or factor loadings (λ_*i*_). Also, the latent variable θ may be manifested as varying types of relevantly formed compilation of items such as a raw score (θ*_*X*_*), factor score variable (θ*_*FA*_*), principal component score variable (θ*_*PC*_*), a theta score formed by the item response theory (IRT) or Rasch modeling (θ*_*IRT*_*), or a possible non-linear compilation of the items (θ*_*NonL*_*).

**FIGURE 2:**
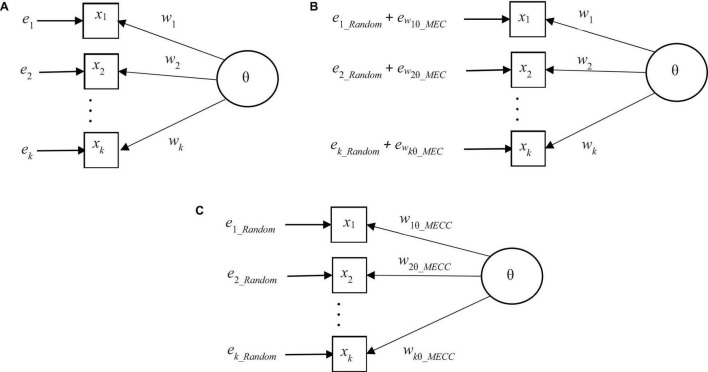
**(A)** A general one-factor measurement model without elements of related to deflation. **(B)** A general one-factor measurement model with elements of error related to deflation. **(C)** Deflation-corrected one-latent variable measurement model.

Eq. (5) generalizes to the compilation of items as


(6) $$\sum_{i=1}^{k}x_{i}=\sum_{i=1}^{k}w_{i}\,\theta +\sum_{i=1}^{k}e_{i},$$

where *k* is the number of items in the compilation. Eq. (6) corresponds with the classic relation of the observed score (*X*), true score (*T*), and error (*E*) in the classical measurement model, that is, *X* = *T* + *E* discussed above. To visualize the differences between different models, this general (congeneric, one-latent variable) model without considering the elements of deflation is as in Figure 2A.

From the correlation viewpoint, knowing that all generally used estimators of correlation give identical estimates of the correlation for original variables and for the standardized versions of the variables, without loss of generality, we can assume that *g*_*i*_ and θ are standardized, *x*_*i*_,θ∼*N*(0,1). Then, parallel to the traditional model (see e.g., Cheng et al., 2012), the error variance of the test score $\psi_{i}^{2}$ can be estimated as


(7) $$\psi_{i}^{2}=\sigma_{E}^{2}=V\,A\,R\,\left(\sum_{i=1}^{k}e_{i}\right)=\sum_{i=1}^{k}\left(1-w_{i}^{2}\right).$$

Eq. (7) can be strictly used in estimating the reliability of the score variable (*REL* = $1-\sigma_{E}^{2}\,/\,\sigma_{X}^{2}$). If we use principal component loadings as the weight factor and principal component score as a manifestation of θ, the conceptualization of error variance in Eq. (7) is used strictly in *ρ_*TH*_* (Eq. 2) and, when using factor loadings and factor score variable, it leads to such estimators as *ρ_ω_* and *ρ_*MAX*_* (Eqs. 3 and 4).

The traditional estimators of reliability assume that *Rit* and factor/principal component loadings are deflation-free. This is a too optimistic assumption as discussed and illustrated above (see Figure 1). If the observed value of *w*_*i*_ embeds deflation, as it typically does when using the traditional estimators of correlation and loadings, the magnitude of the observed correlation or loading by a deflated or MEC-defected (MECD) weight factor (*w*_*i_ MECD*_) is, obviously, lower than MEC-free (MECF) weight factor (*w*_*i*_*MECF*_), that is,


(8a) wi=_⁢M⁢E⁢C⁢Fwi⁢_⁢M⁢E⁢C⁢D+ew⁢i⁢_⁢M⁢E⁢C

or


(8b) $$w_{i\,\_\,M\,E\,C\,D}=w_{i\,\_\,M\,E\,C\,F}-e_{w\,i\,\_\,M\,E\,C}$$

where the exact magnitude of the error element related to deflation in estimation (*e*_*wi_ MEC*_) is largely unknown although it is positive (*e*_*wi_ MEC*_ > 0), and it depends on the characteristics of the item and the weight factor as discussed above. While knowing that a certain part of the measurement error is strictly technical or mechanical in nature, but its magnitude could be reduced, it makes sense to reconceptualize the classic relation of *X* = *T* + *E* into a form


(9) $$X=T+\left(E_{R\,a\,n\,d\,o\,m}+E_{M\,E\,C}\right),$$

where the element *E*_*MEC*_ related to deflation is something we can deal with. Notably, this kind of “systematic error” is not a kind we usually consider as “systematic” such as a typo in the test item or some technical problem in processes (see Gulliksen, 1950; Krippendorff, 1970). The latter type of error is usually considered harmless from the reliability viewpoint and its effect is added to the random part of the error. Consequently, we can reconceptualize the measurement model in Eq. (5) as


(10) $$x_{i}=w_{i}\times \theta +\left(e_{i\,\_\,R\,a\,n\,d\,o\,m}+e_{w\,i\,\theta \,\_\,M\,E\,C}\right),$$

where the notation *e*_*wiθ _ MEC*_refers to the fact that the magnitude of the deflation depends on the characteristics of the weighting factor *w*, item *i*, and the score variable θ. This model using a weight factor including radical deflation such as *Rit* or *λ_*i*_* may be illustrated as in Figure 2B. Notably, the magnitude of the total error (*e*_*i*_*Random*_ + *e*_*wi*θ_*MEC*_) is, factually, equal to the one seen in the model in Figure 2A. However, now the two components are just visual.

While knowing that some estimators of correlation are less deflated than some others, it makes sense to select such coefficient as the weighting factor where the quantity of technical or mechanical error would be as low as possible. However, it may be difficult to find an estimator of correlation without deflation, that is, that would be totally deflation- or MEC-free. In what follows, the concept of deflation-*corrected* and, specifically, MEC-*corrected* estimator (MECC) is used to refer such estimators where the deflation is known to be radically smaller than in PMC. If selecting wisely the weight factor, the magnitude of error component related to deflation may be near zero, that is, *e*_*wi*θ_*MEC*_≈0. If we use options of *w*_*i*_ that would lead us to the condition of *e*_*wi*θ_*MEC*_≈0, because of Eq. (10), this will lead us to a model where the measurement error would be as near the MEC-free condition as possible, that is,


(11) $$x_{i}=w_{i\,\_\,M\,E\,C\,C}\times \theta +\left(e_{i\,\_\,R\,a\,n\,d\,o\,m}+e_{w\,i\,\theta \,\_\,M\,E\,C}\right)\approx w_{i\,\_\,M\,E\,C\,C}\times \theta +e_{i\,\_\,R\,a\,n\,d\,o\,m}.$$

This measurement model where MEC-corrected weight factors such as *RPC*, *G*, or *D* are used, could be illustrated as in Figure 2C.

As with Eq. (7), knowing that all generally used estimators of correlation give identical estimate of the correlation for original variables (*g*_*i*_ and θ) and for the standardized versions of the variables, we can assume that *g*_*i*_ and θ are standardized, *x*_*i*_,θ∼*N*(0,1). Then, assuming that item-wise random errors do not depend on the true scores, the item-wise MEC-corrected error variance ($\psi_{i\,\_\,M\,E\,C\,C}^{2}$) is


(12) $$\psi_{i\,\_\,M\,E\,C\,C}^{2}=V\,A\,R\,\left(e_{i}\right)=V\,A\,R\,\left(x_{i}\right)-{\left(w_{i\,\_\,M\,E\,C\,C}\right)}^{2}\times V\,A\,R\,\left(\theta \right)=1-w_{i\,\_\,M\,E\,C\,C}^{2},$$

that is, $e_{i\,\_\,M\,E\,C\,C}∼N\,\left(0,\psi_{i\,\_\,M\,E\,C\,C}^{2}\right)$ where $\psi_{i\,\_\,M\,E\,C\,C}^{2}=1-w_{i\,\_\,M\,E\,C\,C}^{2}$. Then, after the deflation-correction, the Eq. (9) could be written as


(13) $$X=T+E_{R\,a\,n\,d\,o\,m}+E_{M\,E\,C}-E_{M\,E\,C}=T+E_{R\,a\,n\,d\,o\,m}$$

and Eq. (10) as


(14) $$\sum_{i=1}^{k}x_{i}=\sum_{i=1}^{k}w_{i\,\_\,M\,E\,C\,C}\times \theta +\sum_{i=1}^{k}e_{i\,\_\,R\,a\,n\,d\,o\,m}.$$

Consequently, the deflation-corrected error variance of the test score can be written as


(15) $$\sum_{i=1}^{k}\psi_{i\,\_\,M\,E\,C\,C}^{2}=\sum_{i=1}^{k}\left(1-w_{i\,\_\,M\,E\,C\,C}^{2}\right),$$

where the form corresponds to the traditional error variance


(16) $$\sum_{i=1}^{k}\psi_{i}^{2}=\sum_{i=1}^{k}\left(1-\lambda_{i}^{2}\right)$$

used in the traditional estimators of omega and rho in Eqs. (3) and (4) (see, e.g., Cheng et al., 2012). In the deflation-corrected estimators or reliability, instead of using factor- or principal component loadings we use deflation-corrected estimators of correlation.

### Theoretical Deflation-Corrected Estimators of Reliability

By being open for different manifestations of *w*_*i*_ and θ, some options for the base of the deflation-corrected estimators of reliability are theoretical deflation-corrected alpha based on Eq. (1):


(17) ρα=_⁢w⁢i⁢θkk-1(1-∑i=1kσi2(∑i=1kσi×wi⁢θ)2),

theoretical deflation-corrected theta based on Eq. (2):


(18) $$\rho_{T\,H\,\_\,w\,i\,\theta }=\frac{k}{k-1}\,\left(1-\frac{1}{\sum_{i=1}^{k}w_{i\,\theta }^{2}}\right),$$

theoretical deflation-corrected omega based on Eq. (3):


(19) $$\rho_{\omega \,\_\,w\,i\,\theta }=\frac{{\left(\sum_{i=1}^{k}w_{i\,\theta }\right)}^{2}}{{\left(\sum_{i=1}^{k}w_{i\,\theta }\right)}^{2}+\sum_{g=1}^{k}\left(1-w_{i\,\theta }^{2}\right)},$$

and theoretical deflation-corrected rho based on Eq. (4):


(20) $$\rho_{M\,A\,X\,\_\,w\,i\,\theta }=\frac{1}{1+\frac{1}{\sum_{i=1}^{k}\left(w_{i\,\theta }^{2}\,/\,\left(1-w_{i\,\theta }^{2}\right)\right)}},$$

where *w*_*iθ*_ refers to the general model where the manifestations of θ may vary as well as the linking coefficient *w* and, obviously, the estimate varies item-wise. Obviously, using the estimators (17) to (20) outside of their original context of raw scores or principal component- and factor analysis is debatable. Here, a stand-point is taken that the forms *could* be used as stand-alone estimators even without their original contexts. This is consistent with a more general measurement model discussed above. Alternatively, the estimators (18) to (20) may be taken as an output of renewed procedures in the principal component- and factor analysis where *w*_*i*_ is a less deflated estimator of correlation than the traditional principal component- and factor loading.

### Examples of Practical Deflation-Corrected Estimators of Reliability

By combining the theoretical estimators in Eqs. (17) to (20) and different operationalizations of *w*_*i*_, we get varying families of deflation-corrected estimator of reliability. Let us assume that we do not fix the manifestation of θ, and we use such MEC-corrected weight factors as *RPC*, *G* and *D* directed so that “item given score” or *D* = *D*(*i*|*X*) usually labeled as “score dependent” in the common software packages (of the correct direction of *D*, see Metsämuuronen, 2020b). This leads us to such practical family of deflation-corrected estimators of reliability as deflation-corrected alpha based on Eq. (17) as


(21) ρα=_⁢R⁢P⁢C⁢i⁢θkk-1(1-∑i=1kσi2(∑i=1kσi×R⁢P⁢Ci⁢θ)2),

(22) ρα=_⁢G⁢i⁢θkk-1(1-∑i=1kσi2(∑i=1kσi×Gi⁢θ)2),

and


(23) ρα=_⁢D⁢i⁢θkk-1(1-∑i=1kσi2(∑i=1kσi×D⁢(g|θ)i⁢θ)2)=kk-1⁢(1-∑σi2i=1k(∑i=1kσi×Di⁢θ)2).

Because of using totally different type of estimator than PMC, these could be called special types of DCERs, namely, MEC-corrected estimators of reliability. If using some relevant attenuation-corrected estimator of correlation (see some options in Metsämuuronen, 2021c), a family of attenuation-corrected alpha would be obtained.

The notation in names ρ_α_*RPCi*θ_, ρ_α_*Gi*θ_, and ρ_α_*Di*θ_ refers to the facts that the base of the estimator is alpha (α), the weight factor is manifested as *RPC*, *G*, or *D* representing different types of correlations between item and the score variable, and the manifestation of the score variable (θ) could be a raw score (θ*_*X*_*) or factor score variable (θ*_*FA*_*), as examples. Some of these kinds of estimators are discussed by Metsämuuronen and Ukkola (2019) and Metsämuuronen (2020b,2021a,2021b). Another type of solution is discussed by Zumbo et al. (2007) and Gadermann et al. (2012) by replacing the matrix of PMCs by a matrix of *RPC*s in forming the factor loadings; this leads to a coefficient called ordinal alpha discussed above.

More effective estimators than above are expected if coefficient theta (Eq. 18) is used as a base for the estimators and

*RPC*, *G*, and *D* as *w*_*i*_.^4^ We get a family of deflation-corrected theta based on Eq. (18):


(24) $$\rho_{T\,H\,\_\,R\,P\,C\,i\,\theta }=\frac{k}{k-1}\,\left(1-\frac{1}{\sum_{i=1}^{k}R\,P\,C_{i\,\theta }^{2}}\right),$$

(25) $$\rho_{T\,H\,\_\,G\,i\,\theta }=\frac{k}{k-1}\,\left(1-\frac{1}{\sum_{i=1}^{k}G_{i\,\theta }^{2}}\right),$$

and


(26) $$\rho_{T\,H\,\_\,D\,i\,\theta }=\frac{k}{k-1}\,\left(1-\frac{1}{\sum_{i=1}^{k}D_{i\,\theta }^{2}}\right)$$

or a family of deflation-corrected omega based on Eq. (19):


(27) $$\rho_{\omega \,\_\,R\,P\,C\,i\,\theta }=\frac{{\left(\sum_{i=1}^{k}R\,P\,C_{i\,\theta }\right)}^{2}}{{\left(\sum_{i=1}^{k}R\,P\,C_{i\,\theta }\right)}^{2}+\sum_{i=1}^{k}\left(1-R\,P\,C_{i\,\theta }^{2}\right)},$$

(28) $$\rho_{\omega \,\_\,G\,i\,\theta }=\frac{{\left(\sum_{i=1}^{k}G_{i\,\theta }\right)}^{2}}{{\left(\sum_{i=1}^{k}G_{i\,\theta }\right)}^{2}+\sum_{i=1}^{k}\left(1-G_{i\,\theta }^{2}\right)},$$

and


(29) $$\rho_{\omega \,\_\,D\,i\,\theta }=\frac{{\left(\sum_{i=1}^{k}D_{i\,\theta }\right)}^{2}}{{\left(\sum_{i=1}^{k}D_{i\,\theta }\right)}^{2}+\sum_{i=1}^{k}\left(1-D_{i\,\theta }^{2}\right)},$$

or a family of deflation-corrected rho based on Eq. (20):


(30) $$\rho_{M\,A\,X\,\_\,R\,P\,C\,i\,\theta }=\frac{1}{1+\frac{1}{\sum_{i=1}^{k}\left(R\,P\,C_{i\,\theta }^{2}\,/\,\left(1-R\,P\,C_{i\,\theta }^{2}\right)\right)}},$$

(31) $$\rho_{M\,A\,X\,\_\,G\,i\,\theta }=\frac{1}{1+\frac{1}{\sum_{i=1}^{k}\left(G_{i\,\theta }^{2}\,/\,\left(1-G_{i\,\theta }^{2}\right)\right)}},$$

and


(32) $$\rho_{M\,A\,X\,\_\,D\,i\,\theta }=\frac{1}{1+\frac{1}{\sum_{i=1}^{k}\left(D_{i\,\theta }^{2}\,/\,\left(1-D_{i\,\theta }^{2}\right)\right)}}.$$

These families could be called also MEC-corrected theta, omega, and rho. Notably, Zumbo et al. (2007) and Gadermann et al. (2012) also discuss the use of Armor’s theta as a basis for ordinal theta by replacing the matrix of PMCs by a matrix of *RPC*s in the estimation.

Many good or even better alternative could be found for *RPC*, *G*, and *D* considering that using *RPC* may lead us to challenges in interpreting the reliability as reflecting unobservable variables (see critique in Chalmers, 2017) and *G* tend to underestimate correlation when there are more than four categories in the item and *D* with three categories or more (see Metsämuuronen, 2021b). For the polytomous case, instead of *G* and *D*, the dimension-corrected *G* and *D* are suggested (Metsämuuronen, 2021b).

The characteristics of the estimators above are not discussed in-depth here; simulations would be beneficial in this matter. However, in the hypothetic extreme datasets with deterministic item discrimination in *all* items leading to *RPC*_*i*_ = *RPC*_*j*_ ≈ *G*_*i*_ = *G*_*j*_ = *D*_*i*_ = *D*_*j*_ = 1,^5^ DCERs based on theta and omega would lead to perfect reliability: ρ_*TH*_*RPCi*θ_≈ρ_*TH*_*Gi*θ_ = ρ_*TH*_*Di*θ_ = *k*/(*k*−1)(1−1/*k*)≡1 and ρ_ω_*RPCi*θ_≈ρ_ω_*Gi*θ_ = ρ_ω_*Di*θ_ = (*k*)^2^/((*k*)^2^ + 0)≡1. In the case, estimators (21) to (23) based on alpha can reach the value ρ_α_*RPCi*θ_≈ρ_α_*Gi*θ_ = ρ_α_*Di*θ_ = 1 only when all item variances are equal (*σ_*i*_* = *σ_*i*_* = σ), that is, for instance, when the items are standardized. In the case, ρα⁢_=R⁢P⁢C⁢i⁢θρα⁢_=G⁢i⁢θρα⁢_=D⁢i⁢θ*k*/(*k*−1)(1−*k*σ^2^/(*k*(σ×1))^2^) = *k*/(*k*−1)×(1−1/*k*)≡1. Otherwise, the maximum value is $\rho_{\alpha \,\_\,R\,P\,C\,i\,\theta }^{M\,a\,x}\approx \rho_{\alpha \,\_\,G\,i\,\theta }^{M\,a\,x}=\rho_{\alpha \,\_\,D\,i\,\theta }^{M\,a\,x}=\frac{k}{k-1}\,\left(1-\sum_{i=1}^{k}\sigma_{i}^{2}\,/\,{\left(\sum_{i=1}^{k}\sigma_{i}\right)}^{2}\right)$. Notably, in the deterministic case, estimators based on rho (Eqs. 30 to 32) could not be used because this would require division by zero which is not defined. Aquirre-Urreta et al. (2019) also noted that rho may produce overestimates of the true reliability with finite samples familiar in real-world testing settings. A practical reason for this is that the formula is sensitive to very high values of loadings. In small sample sizes familiar in the real-world datasets, the possibility to obtain deterministic or near-deterministic situation in one or several items increases. In deterministic patterns, *ρ*_*MAX*_ cannot be estimated at all and in the near-deterministic patterns the factor loading may be artificially high leading to obvious overestimation in reliability. In what follows in a numerical example, the outcomes based on the DCERs in Eqs. (21) to (23), (30) and (31) are illustrated and the traditional estimators (1) to (4) are used as benchmarks.

## Materials and Methods

### Dataset Used in the Numerical Example

As a simple numerical example, the dataset consisting of a set of 30 multiple choice questions forming 30 binary items and *n* = 49 randomly selected test-takers from a national level datasets of mathematics test (*N* = 4,023; FINEEC, 2018) representing small-scale tests with finite samples is used in illustrating the difference between the traditional estimators and deflation-corrected estimators of reliability. The dataset with estimates of different score variables and weight factors are in Supplementary Appendix 1.^6^

### Measurement Model

The general measurement model discussed in section “Conceptual Base of the Deflation-Corrected Estimators of Reliability” is applied. By using the general one-factor model and by varying *w* and the operationalization of θ, examples of traditional and deflation-corrected estimates of reliability of the score are given by modifying mainly the form of rho (Eq. 20) with some benchmarking estimates by the form of alpha (Eq. 17).

### Operationalizations of the Latent Variable and the Linking Factor

In the empirical section, five operationalizations for θ are used: an unweighted raw score (θ_*X*_), a principal component score variable (θ_*PC*_), a factor score variable by maximum likelihood estimation (θ_*FA*_), a theta score by one-parameter IRT model or Rasch model (θ_*IRT*_), and a nonlinear weighted score by a simple weighting factor 1/*p*_*i*_ ($\theta_{N\,o\,n\,L}=\theta_{P\,I}=\sum_{i=1}^{k}g_{i}\,/\,p_{i}$) where the test-takers are weighted by the proportion of correct answers *p*_*i*_; the more demanding item, the higher the weight.

Seven options as the weighting factor between θ and *g*_*i*_ are used. First, traditional estimators used in the traditional estimators of reliability: *Rit* with θ_*X*_, principal component loading with θ_*PC*_, and ML-estimate of the factor loading with θ_*FA*_; second, alternative coefficients *RPC, G*, and *D* for deflation-corrected estimators of reliability; and, third, the traditional PMC (later, *R* or *R*_*i*θ_) as a benchmarking coefficient for the DCERs when not using the traditional alpha. The statistics for and calculations of the estimates are collected in Supplementary Appendix 1.

Combining the operationalizations above, we get estimators of reliability related to five different scores and seven linking factors; only selected combinations are used (see condensed in Table 1).

**TABLE 1:** Estimators of reliability covered in the empirical section.

	Weight factor (the base of the estimator)										
		*Rit(alpha)^a^*	*RPC(alpha)^b^*	*G(alpha)^b^*	*D(alpha)^b^*	λ_*PC*_*(theta)^a^*	λ_*ML*_*(omega)^a^*	λ_*ML*_*(rho)^a^*	*R(rho)^b^*	*RPC(rho)^b^*	*G(rho)^b^*
	Eqs.	1	21	22	23	2	3	4, 33	34–38	39–43	44–48
Score type	*θ_*X*_*	x	x	x	x				x	x	X
	*θ_*PC*_*					x			x	x	X
	*θ_*FA*_*						x	x	x	x	X
	*θ_*IRT*_*								x	x	X
	*θ_*PI*_*								x	x	X

*^a^Traditional estimates.*

*^b^Deflation-corrected estimates.*

First, traditional estimators (alpha, theta, omega, and rho; Eqs. 1–4) of which rho is re-notated here to match with the other estimators:


(33) $$\rho_{M\,A\,X\,\_\,\lambda \,i\,\theta_{F\,A}}=\rho_{M\,A\,X}=\frac{1}{1+1\,/\,\sum_{i=1}^{k}\left(\lambda_{i\,\theta_{F\,A}}^{2}\,/\,\left(1-\lambda_{i\,\theta_{F\,A}}^{2}\right)\right)},$$

where the notation *ρ*_*MAX_ λiθ _FA*_refers to facts that coefficient rho is the base of the coefficient (*MAX*), the manifestation of the score variable is the factor score variable ($\theta_{F\,A}$), and the manifestation of the weight factor is the ML-estimate of the factor loading (*w_*i*__=__λ_i =λ_iθFA_*).

Second, five estimators based on the form of rho and item–score correlation (ρ_*i*θ_ = *R*_*i*θ_) as the linking factor:


(34) $$\rho_{M\,A\,X\,\_\,R\,i\,\theta_{X}}=\frac{1}{1+1\,/\,\sum_{i=1}^{k}\left(R_{i\,\theta_{X}}^{2}\,/\,\left(1-R_{i\,\theta_{X}}^{2}\right)\right)},$$

where the score is θ_*X*_ and *w*_*i*_ = *R*_*iθX*_, (34)


(35) $$\rho_{M\,A\,X\,\_\,R\,i\,\theta_{P\,C}}=\frac{1}{1+1\,/\,\sum_{i=1}^{k}\left(R_{i\,\theta_{P\,C}}^{2}\,/\,\left(1-R_{i\,\theta_{P\,C}}^{2}\right)\right)},$$

where the score is θ_*PC*_ and *w*_*i*_ = *R*_*iθPC*_,


(36) $$\rho_{M\,A\,X\,\_\,R\,i\,\theta_{F\,A}}=\frac{1}{1+1\,/\,\sum_{i=1}^{k}\left(R_{i\,\theta_{F\,A}}^{2}\,/\,\left(1-R_{i\,\theta_{F\,A}}^{2}\right)\right)},$$

where the score is θ_*FA*_ and *w*_*i*_ = *R*_*iθFA*_,


(37) $$\rho_{M\,A\,X\,\_\,R\,i\,\theta_{I\,R\,T}}=\frac{1}{1+1\,/\,\sum_{i=1}^{k}\left(R_{i\,\theta_{I\,R\,T}}^{2}\,/\,\left(1-R_{i\,\theta_{I\,R\,T}}^{2}\right)\right)},$$

where the score is θ_*IRT*_ and *w*_*i*_ = *R*_*iθIRT*_, and


(38) $$\rho_{M\,A\,X\,\_\,R\,i\,\theta_{P\,I}}=\frac{1}{1+1\,/\,\sum_{i=1}^{k}\left(R_{i\,\theta_{P\,I}}^{2}\,/\,\left(1-R_{i\,\theta_{P\,I}}^{2}\right)\right)},$$

where the score is θ_*PI*_ and *w*_*i*_ = *R*_*iθPI*_.

Third, the parallel estimators using *RPC* = *RPC*_*iθ*_ as the linking factor:


(39) $$\rho_{M\,A\,X\,\_\,R\,P\,C\,i\,\theta_{X}}=\frac{1}{1+1\,/\,\sum_{i=1}^{k}\left(R\,P\,C_{i\,\theta_{X}}^{2}\,/\,\left(1-R\,P\,C_{i\,\theta_{X}}^{2}\right)\right)},$$

where the score is θ_*X*_ and *w*_*i*_ = *RPC*_*iθX*_,


(40) $$\rho_{M\,A\,X\,\_\,R\,P\,C\,i\,\theta_{P\,C}}=\frac{1}{1+1\,/\,\sum_{i=1}^{k}\left(R\,P\,C_{i\,\theta_{P\,C}}^{2}\,/\,\left(1-R\,P\,C_{i\,\theta_{P\,C}}^{2}\right)\right)},$$

where the score is θ_*PC*_ and *w*_*i*_ = *RPC*_*iθPC*_,


(41) $$\rho_{M\,A\,X\,\_\,R\,P\,C\,i\,\theta_{F\,A}}=\frac{1}{1+1\,/\,\sum_{i=1}^{k}\left(R\,P\,C_{i\,\theta_{F\,A}}^{2}\,/\,\left(1-R\,P\,C_{i\,\theta_{F\,A}}^{2}\right)\right)},$$

where the score is θ_*FA*_ and *w*_*i*_ = *RPC*_*iθFA*_,


(42) $$\rho_{M\,A\,X\,\_\,R\,P\,C\,i\,\theta_{I\,R\,T}}=\frac{1}{1+1\,/\,\sum_{i=1}^{k}\left(R\,P\,C_{i\,\theta_{I\,R\,T}}^{2}\,/\,\left(1-R\,P\,C_{i\,\theta_{I\,R\,T}}^{2}\right)\right)},$$

where the score is θ_*IRT*_ and *w*_*i*_ = *RPC*_*iθIRT*_, and


(43) $$\rho_{M\,A\,X\,\_\,R\,P\,C\,i\,\theta_{P\,I}}=\frac{1}{1+1\,/\,\sum_{i=1}^{k}\left(R\,P\,C_{i\,\theta_{P\,I}}^{2}\,/\,\left(1-R\,P\,C_{i\,\theta_{P\,I}}^{2}\right)\right)},$$

where the score is θ_*PI*_ and *w*_*i*_ = *RPC*_*iθPI*_.

Fourth, the parallel estimators using *G = G_*i*_*_θ_ as the linking factor:


(44) $$\rho_{M\,A\,X\,\_\,G\,i\,\theta_{X}}=\frac{1}{1+1\,/\,\sum_{i=1}^{k}\left(G_{i\,\theta_{X}}^{2}\,/\,\left(1-G_{i\,\theta_{X}}^{2}\right)\right)},$$

where the score is $\theta_{X}$ and *w*_*i*_ = *G*_*iθX*_,


(45) $$\rho_{M\,A\,X\,\_\,G\,i\,\theta_{P\,C}}=\frac{1}{1+1\,/\,\sum_{i=1}^{k}\left(G_{i\,\theta_{P\,C}}^{2}\,/\,\left(1-G_{i\,\theta_{P\,C}}^{2}\right)\right)},$$

where the score is θ_*PC*_ and *w*_*i*_ = *G*_*iθPC*_,


(46) $$\rho_{M\,A\,X\,\_\,G\,i\,\theta_{F\,A}}=\frac{1}{1+1\,/\,\sum_{i=1}^{k}\left(G_{i\,\theta_{F\,A}}^{2}\,/\,\left(1-G_{i\,\theta_{F\,A}}^{2}\right)\right)},$$

where the score is θ_*FA*_ and *w*_*i*_ = *G*_*iθFA*_,


(47) $$\rho_{M\,A\,X\,\_\,G\,i\,\theta_{I\,R\,T}}=\frac{1}{1+1\,/\,\sum_{i=1}^{k}\left(G_{i\,\theta_{I\,R\,T}}^{2}\,/\,\left(1-G_{i\,\theta_{I\,R\,T}}^{2}\right)\right)},$$

where the score is θ_*IRT*_ and *w*_*i*_ = *G*_*iθIRT*_,

and


(48) $$\rho_{M\,A\,X\,\_\,G\,i\,\theta_{P\,I}}=\frac{1}{1+1\,/\,\sum_{i=1}^{k}\left(G_{i\,\theta_{P\,I}}^{2}\,/\,\left(1-G_{i\,\theta_{P\,I}}^{2}\right)\right)},$$

where the score is θ_*PI*_ and *w*_*i*_ = *G*_*iθ _PI*_.

Additionally, DCERs based on coefficient alpha (Eqs. 21–23) are used as benchmarks to the traditional estimators (see Table 1). Of the calculation of the estimates, see Supplementary Appendix 1.

## Results

**FIGURE 3:**
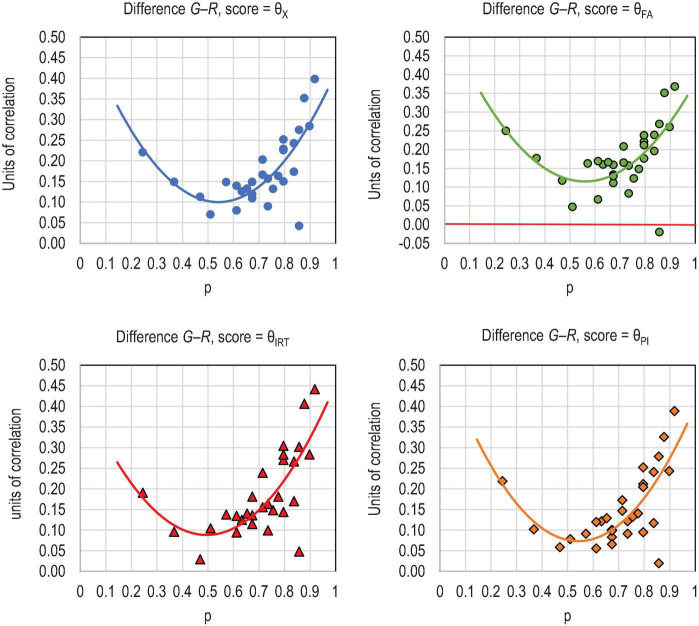
Difference between the estimates of item–score correlation by G and PMC (R).

Eight outcomes of the comparison are worth highlighting. First, of the estimators based on the form of rho (Eqs. 33 to 48), the ones using *RPC* and *G* as the linking factor give notably higher estimates (0.961–0.968) in comparison to those using PMC (0.894–0.909) and traditional factor- or principal component loadings (ρ_*MAX*_ = 0.894, ρ_ω_ = 0.864, ρ_*TH*_ = 0.879) or alpha (*ρ*_α_ = 0.862) (Table 2). This is caused by the better behavior of *RPC* and *G* in relation to deflation with the items with extreme difficulty levels in comparison to PMC (see Figure 3). The estimates of reliability based on *RPC* and *G* tend to be more deflation-free than those based on traditional principal component- and factor loadings or PMC, that is, *e*_*Rit_MEC*,_ *e_λ*i_*_*_*MEC*_ > > *e*_*RPCi*θ_*___*_*MEC*_≈ *e*_*Gi*θ_*___*_*MEC*_*.* The possible overestimation by DCERs is discussed later.

**TABLE 2:** Comparison of the estimates of reliability.

	Weight factor (the base of the estimator)										
		*Rit(alpha)^a^*	*RPC(alpha)^b^*	*G(alpha)^b^*	*D(alpha)^b^*	λ_*PC*_*(theta)^a^*	λ_*ML*_*(omega)^a^*	λ_*ML*_*(rho)^a^*	*R(rho)^b^*	*RPC(rho)^b^*	*G(rho)^b^*
	Eqs.	1	21	22	23	2	3	4, 33	34–38	39–43	44–48
Score type	*θ_*X*_*	0.8619	0.9374	0.9420	0.9343				0.9024	0.9628	0.9682
	*θ_*PC*_*					0.8789			0.9069	0.9661	0.9656
	*θ_*FA*_*						0.8641	0.8943	0.9094	0.9688	0.9681
	*θ_*IRT*_*								0.8944	0.9628	0.9682
	*θ_*PI*_*								0.8987	0.9614	0.9609

*^a^Traditional estimates.*

*^b^Deflation-corrected estimates.*

Second, in comparison to the estimates by Eqs. (34) to (38) related to PMC (0.894–0.909) and the traditional *ρ*_*MAX*_ (0.894), the estimates by Eqs. (39) to (48) related to *RPC* and *G* tend to be close to each other (0.961–0.969) even though they indicate different aspects of the correlation. While *RPC* estimates the inferred correlation of the (unobservable) latent variables, G estimates the probability that the test takers are in the same order both in an item and a score. The same magnitude of the estimates may be interpreted to indicate that the estimators reflect the same deflation-free reliability of the test score.

Third, the magnitudes of the estimates by the traditional coefficients rho by Eq. (4)(ρ_*MAX*_λ*i*θ_*FA*__ = ρ_*MAX*_ = 0.894), theta by Eq. (2) (ρ_*TH*_ = 0.879), and omega by Eq. (3) (ρ_ω_ = 0.864) are higher than by the traditional coefficient alpha by Eq. (1) (*ρ*_α_ = 0.862). This is expected because only in the theoretical case that all the factor loadings or item–score correlations are equal, the magnitude of the estimates by *ρ*_α_ would reach those by the other estimates. However, it seems that *ρ*_*MAX*_ does not produce the “maximal” reliability *per se* for the given test. In the dataset at hand, even the traditional PMC between an item and the factor score variable would lead to a somewhat higher estimate (ρ_*MAX*_*Ri*θ_*FA*__ = 0.909) than using the factor loadings nothing to say of the deflation-corrected estimates (ρ_*MAX*_*RPCi*θ_*FA*__ = 0.969 and ρ_*MAX*_*Gi*θ_*FA*__ = 0.968). Hence, the thinking that “maximal reliability (in the form seen in Eq. 4) is the highest possible reliability that a test can achieve” (Cheng et al., 2012, p. 53 as an example), seems not be true in the absolute sense. Notably though, when using PMC and *RPC* as the linking factor, the score formed by the factor modeling, traditionally taken as the “optimal linear combination” of the items (see, Li, 1997), tends to have the highest reliability in comparison to the other types of score variables although the difference is not notable.

Fourth, coefficient alpha is known to underestimate the true reliability. By using the DCERs based on alpha, that is, Eqs. (21) to (23), the estimates are notably higher (ρα=_⁢R⁢P⁢C⁢i⁢θX0.937, ρα=_⁢G⁢i⁢θX0.942, and ρα=_⁢D⁢i⁢θX0.934), and these are not far from the estimates by the DCERs based on rho with the raw score ρ_*MAX*_*RPCi*θ_*X*__ = 0.963 by Eq. (39) and ρ_*MAX*_*Gi*θ_*X*__ = 0.968 by Eq. (44). This seems to indicate that the reliability of the raw score may be closer than what we have thought to the ones manifested as the optimal linear combination of the items.

Fifth, obviously, the outcomes of forming the score differ radically from each other. On the one hand, the scores formed by PCA, EFA, and IRT modeling follow the standardized normal distribution while the raw score and the non-linearly weighted score differ from this logic. On the other hand, the score variables by PCA (θ*_*PC*_*), EFA (θ*_*FA*_*), and non-linear summing (θ*_*PI*_*) do not include tied cases in the dataset; each test takers got their own category in θ*_*PC*_*, θ*_*FA*_* and θ*_*PI*_* while the scores by IRT (θ*_*IRT*_*) and the raw score (θ*_*X*_*) have identical number of tied cases; in the one-parameter model used in the analysis, θ*_*IRT*_* is a logistic transformation of θ*_*X*_*. Consequently, the DCERs for the raw score (Eqs. 39 and 44) and for the IRT score (Eqs. 42 and 47) are identical (ρ_*MAX*_*RPCi*θ_*X*__ = ρ_*MAX*_*RPCi*θ_*IRT*__ = 0.963 and ρ_*MAX*_*Gi*θ_*X*__ = ρ_*MAX*_*Gi*θ_*IRT*__ = 0.968) because the order of the test takers remains the same in the logistic transformation. Regardless of the differences in the structure of the score variables, the estimators based on *G* as a linking factor produce estimates that are largely at the same magnitude of reliability with the scores by raw score, EFA, and IRT by Eqs. (44), (46), and (47): ρ_*MAX*_*Gi*θ_*X*__≈ρ_*MAX*_*Gi*θ_*FA*__≈ρ_*MAX*_*Gi*θ_*IRT*__≈0.968 and the differences are not wide either when using *RPC* (0.963–0.969). Notably, when using *RPC* and *G* as the linking factor, the score formed by EFA with no tied cases cannot discriminate the test-takers remarkably more accurately than the score with tied cases (θ*_*IRT*_* or θ*_*X*_*). This reflects the non-obvious fact that reliability of the score variable, in a sense of discriminating the test takers from each other, is not strictly connected with the number of tied values in the score variable nor the type of scale.

Sixth, the obvious reason for the higher magnitude of the estimates by DCERs using *RPC* and *G* in comparison to PMC is caused by the better behavior of *RPC* and *G* with items with extreme difficulty levels. With these kinds of items, specifically, PMC is highly deflated while *RPC* and *G* are not at all affected by item difficulty (see simulation in Metsämuuronen, 2021b). The difference between the estimates of correlation by PMC and *G* is illustrated in Figure 3; the graphs would be essentially identical with PMC and *RPC* because the difference between the estimates by *RPC* and *G* are subtle in binary case (see Metsämuuronen, 2020b,2021b).

Seventh, Green and Yang (2009) approximate that, by using *ρ_α_*, the true reliability may be underestimated up to 11% although, in real-life testing settings, the underestimation may be nominal (Raykov, 1997b). Assuming that *RPC* does not overestimate correlation, when knowing the magnitude of the estimate by the traditional coefficient alpha related to the raw score by Eq. (1) (*ρ_α_* = 0.862) and the deflation-corrected estimate by *RPC* related to the factor score variable by Eq. (33) (ρ_*MAX*_*RPCi*θ_*FA*__ = 0.969) in the given dataset, the magnitude of the deflation in the traditional estimate by *ρ_α_* appears to be 0.1068 units of reliability, that is, 11.0% (=(0.969–0.862)/0.969) in comparison to the one by deflation-corrected rho. By using the same logic, the traditional maximal reliability ρ_*MAX*_ = 0.894 is deflated by 7.7%. These seem decent magnitudes considering that, in the empirical cases, the deflation may be 70 or 44% as discussed in section “Practical Consequences of Mechanical Error in the Estimates of Correlation in Reliability.” The reason for the decent deflation is that the dataset used in the example is neither extremely easy nor extremely difficult. An obvious confounding factor is that the score variables differ between coefficients alpha and rho. If the score variable would be harmonized as being the raw score and the weighting factor would be harmonized to *RPC*, we can assess the pure effect of the estimator itself. The magnitude of the deflation-corrected alpha (Eq. 21) is *ρ_α_*RPCiX*_* = 0.937 and the magnitude of the deflation-corrected rho (Eq. 39) is *ρ_*MAX_RPCiX*_* = 0.963. Then, the deflation would be reduced from 11 to 2.6% (=(0.963–0.937)/0.963). This (around) 3% seems to refer strictly to a more effective estimation of reliability by using the form of estimator based on maximal reliability than by the formula used in the traditional coefficient alpha. Obviously, more studies are needed to confirm the results.

Finally, eighth, by comparing the estimates of different weighting factors *w*_*i*_, it is possible to evaluate roughly what the magnitude of the deflation (*e*_*wiθ _ MEC*_) in different estimators of correlation in the dataset is. Assuming that the estimates by *RPC* do not overestimate the correlation between the items and the score, the difference between the estimates based on *RPC* and PMC gives a hint of the magnitude of the deflation in PMC. On average in the given dataset, the deflation in PMC with different types of score variable is ${\bar{e}}_{R\,i\,\theta_{X}\,\_\,M\,E\,C}=0.156$ units of correlation with raw score (ranging 0.0279–0.3268 depending on the item), ${\bar{e}}_{R\,i\,\theta_{F\,A}\,\_\,M\,E\,C}=0.157$ (–0.0064–0.3121) with the factor score, ${\bar{e}}_{R\,i\,\theta_{I\,R\,T}\,\_\,M\,E\,C}=0.166$ (0.0315–0.3702) with the theta score by IRT modeling, and ${\bar{e}}_{R\,i\,\theta_{P\,I}\,\_\,M\,E\,C}=0.153$(0.0061–0.3433) with the non-linearly weighted score. The systematic negative bias of this size has a notable effect in deflation in the estimate of reliability.

## Conclusion and Limitations

An obvious conclusion of the theoretical and empirical parts of the study is that the magnitude of the deflation of reliability depends not only on the unidimensionality, violations in the measurement model and latent normality, estimator of reliability, and uncorrelated errors as traditionally suggested with coefficient alpha but also on the estimators of correlation used as the linking factor between the latent trait θ and the test items *g*_*i*_. Some linking factors like PMC are more prone to deflation than some other estimators like *RPC*, *G* and *D* as examples and, hence, the estimates by PMC are more deflated than those by *RPC*, *G* and *D*. Because PMC is embedded in the traditional estimators of reliability, the deflation in correlation is inherited to the estimates of reliability. Systematic studies comparing different estimators of correlation and reliability could be beneficial to understand the phenomenon better.

### Options for Correcting the Deflation in Estimators of Reliability

The root challenge related to deflation in the traditional estimators of reliability seems to be the classical definition of reliability based on *variances* ($\sigma_{X}^{2}$, $\sigma_{T}^{2}$, and $\sigma_{E}^{2}$) leading to use PMC in the practical solutions of estimating reliability. If we would start to create a theory concerning reliability by knowing all the deficiencies of PMC we know today, we may be trying to avoid PMC and, consequently, the variances in the process. To rectify this root challenge, it may be beneficial to rethink the definition of reliability from this perspective. Alternative bases to consider for rethinking reliability may be related to, among other, “sufficiency of information” by Smith (2005), or several options within IRT modeling such as “person separation” by Andrich and Douglas (1977), Andrich (1982), and Wright and Masters (1982), or “information function” discussed by, e.g., McDonald (1999), Cheng et al. (2012), and Milanzi et al. (2015). One alternative for defining reliability is discussed briefly here based on Metsämuuronen (2020b) related to the definition of “ultimately discriminating test score.”

Metsämuuronen (2020b) proposes an operational definition of the *ultimate item discrimination* as a condition where the score can predict response pattern of the test-takers in a single item in a deterministic manner. This could be generalized as a theoretical condition for ultimate reliability as being a condition where the score can predict the order (or item response pattern) of the test takers in a deterministic manner *in all items*. This operational definition alone is not very practical when it comes to estimation of the reliability because the deterministic patterns cannot be estimated by using maximum likelihood method, for example. However, this could be a starting point to develop estimators where different types of estimators of item discrimination as well as *a*-parameter in IRT-modeling could be a visible part of the estimator as in Eqs. (21) to (32). Theoretical and empirical work in this area would be beneficial.

While waiting for development of a sound basis for a new way of thinking, defining, and estimating reliability, practical options lead to a kind of new paradigm in the settings related to measurement modeling: the extended families of deflation-corrected estimators of reliability. One set of family, attenuation-corrected estimators of reliability, not discussed in this article, would be obtained if attenuation-corrected estimators of PMC were used instead of PMC in the estimators. Another set of family, MEC-corrected estimators of reliability focused in this article, is obtained if PMC is replaced by a totally different estimator of correlation that would not be deflated at all or where the magnitude of deflation is remarkably smaller than that in PMC. Several new estimators of deflation-corrected estimators were proposed based on using *RPC*, *G* and *D* as examples instead of PMC in some known estimators of reliability.

In the empirical part, it was demonstrated that if *RPC*, *G*, or *D* would be used instead of PMC in some known estimators of reliability, the deflation in reliability would be corrected to a notable extent. Further simulations with different types of datasets, different item types, different weighting factors, and different base of the estimators (e.g., alpha, theta, omega, or rho) would be beneficial in this regard. The estimates by deflation-corrected estimators are not, factually, “real” reliabilities as such. However, they are *closer* to the deflation-free reliability than the traditional estimates. Empirical examples show that, in specific forms of datasets as in very easy or very difficult tests, the estimates by traditional estimators such as coefficient alpha and rho may be deflated 40–70% because of technical reasons. The DCERs discussed in this article are strong with these kinds of datasets and could be used as a benchmark to the traditional estimators.

### Practical Example of Calculating Deflation-Corrected Estimators of Correlations Discussed in This Article

To give a practical example of the DCERs discussed in this article, let us re-analyze the reliability of the extremely easy dataset (*n* = 7,770) by Metsämuuronen and Ukkola (2019) discussed in section “Practical consequences of Mechanical Error in the Estimates of Correlation in reliability.” The advance of DCERs may be notable in these kinds of datasets where the item difficulties are extreme leading to an ultimately non-normal score (see Table 3). Because of ultimately easy items with mainly binary scales combined with a non-normal score variable, the non-parametric coefficients of correlation may be better options than PMC.

**TABLE 3:** Descriptive statistics of the dataset from Metsämuuronen and Ukkola (2019).

Item (g)	N	Maximum	Mean	*p*	*SD*	Score	Freq.	%
g1	7,770	1	0.96	0.96	0.186	3	4	0.1
g2	7,770	1	0.98	0.98	0.126	4	7	0.1
g3	7,770	1	0.99	0.99	0.088	5	6	0.1
g4	7,770	1	0.91	0.91	0.287	6	20	0.3
g5	7,770	2	1.78	0.89	0.610	7	40	0.5
g6	7,770	1	0.98	0.98	0.122	8	141	1.8
g7	7,770	2	1.97	0.985	0.211	9	809	10.4
g8	7,770	2	1.98	0.99	0.169	10	903	11.6
						11	5,840	75.2
							7,770	100.0

#### Deflation-Corrected Alpha

The traditional coefficient alpha uses raw score (θ*_*X*_*) as the manifestation of the latent ability and item–score correlation (*R*_*gX*_) as the weighting element in the calculation. Estimates by alternative coefficients of item–score association are collected in Table 4; their calculation is described in Supplementary Appendix 1. Notably, first, the magnitudes of the estimates by *Rit* (0.38 on average) are remarkably lower than those by *RPC* (0.72), *G* (0.88), and *D* (0.83). This is caused by its poor behavior with items of extreme difficulty level. Second, the magnitude of the estimates by *RPC* is somewhat lower than those by *G* and *D*. This is not a general characteristic of these coefficients. With binary items, the estimates by *G* and *RPC* tend to be very close each other (see, e.g., Metsämuuronen, 2021b), and when the number of categories in the item increases up to four or higher, the probability that two variables are in the same order indicated by *G* (and *D*) tend to be lower than covariation between the two variables indicated by *PMC* and *RPC* and, hence, the estimates would signal that the true correlation is underestimated (see Metsämuuronen, 2021b). Third, that the magnitude of the estimates by *D* are lower than those by *G* is expected because the estimates by *D* are more conservative in comparison with *G* (e.g., Metsämuuronen, 2021a,b).

**TABLE 4:** Item–score correlations and related statistics needed in estimating reliability.

Item (*g*_*i*_)	*R*_*gX*_ *^a^*	*D*_*gX*_ *^a^*	*G*_*gX*_ *^a^*	*RPC*_*gX*_ *^a^*	*$\sigma_{g}^{2}$* = VAR(*g*)	*R*_*gX*_ × *σ_*g*_*	*D*_*gX*_ × *σ_*g*_*	*G*_*gX*_ × *σ_*g*_*	*RPC*_*gX*_ × *σ_*g*_*
g1	0.351	0.791	0.857	0.677	0.035	0.065	0.147	0.160	0.126
g2	0.268	0.779	0.846	0.618	0.016	0.034	0.098	0.107	0.078
g3	0.283	0.858	0.911	0.696	0.008	0.025	0.076	0.080	0.061
g4	0.458	0.789	0.834	0.736	0.082	0.131	0.226	0.239	0.211
g5	0.746	0.952	0.979	0.931	0.372	0.455	0.580	0.597	0.568
g6	0.260	0.766	0.831	0.602	0.015	0.032	0.094	0.102	0.074
g7	0.327	0.832	0.897	0.702	0.045	0.069	0.176	0.189	0.148
g8	0.373	0.877	0.924	0.760	0.028	0.063	0.148	0.156	0.128
				SUM	0.600	0.874	1.546	1.630	1.395

*^a^R, Pearson correlation; D, Somers delta “X dependent”; G, Goodman–Kruskal gamma; RPC, polychoric correlation coefficient.*

Because of Eq. (1), the traditional coefficient alpha gives the estimate: $\rho_{\alpha }=\frac{8}{8-1}\,\left(1-\frac{0.600}{0.874^{2}}\right)=0.245$. The deflation-corrected alpha using *RPC* as the weighting element (Eq. 21) leads to an estimate ρα=_⁢R⁢P⁢C⁢i⁢X88-1(1-0.6001.3952)=0.790, gamma (Eq. 22) to ρα=_⁢G⁢i⁢X88-1(1-0.6001.6302)=0.885, and delta (Eq. 23) to ρα=_⁢D⁢i⁢X88-1(1-0.6001.5462)=0.856. The estimate by the traditional coefficient alpha is radically deflated, 72%, when comparing it to the DCER using *G* as the weighting element ((0.885−0.245)/0.885 = 0.723) and 69% if using *RPC*. We also note that the magnitude of the estimates of reliability follows strictly the general tendency of the magnitudes of the coefficients of correlation: In comparison with the estimate byρ_α_*GiX*_ the estimate by ρ_α_*DiX*_ is conservative.

#### Deflation-Corrected Theta

The traditional coefficient theta uses principal component score (θ_*PC*_) as the manifestation of the latent ability and principal component loadings (*λ_*i*_*) as the weighting element in the calculation. Loadings and corresponding statistics related to alternative estimators are collected in Table 5. Notably, because there appeared to be no tied pairs between the principal component score and items, the estimates by *G* and *D* are identical.

**TABLE 5:** Principal component loadings and related alternative statistics for estimating reliability.

Item (g)	*λ_*iPC*_*	(λ_*iPC*_)^2^	*D*_*g*θ_ *_*PC*_*	(*D*_*g*θ_*_*PC*_*)^2^	*G*_*g*θ_ *_*PC*_*	(*G*_*g*θ_*_*PC*_*)^2^	*RPC*_*g*θ_ *_*PC*_*	(*RPC*_*g*θ_*_*PC*_*)^2^
g1	0.444	0.197	0.937	0.878	0.937	0.878	0.833	0.694
g2	0.429	0.184	0.960	0.922	0.960	0.922	0.837	0.701
g3	0.593	0.352	0.994	0.988	0.994	0.988	0.947	0.897
g4	0.478	0.228	0.892	0.796	0.892	0.796	0.818	0.669
g5	0.207	0.043	0.737	0.543	0.737	0.543	0.647	0.419
g6	0.375	0.141	0.939	0.882	0.939	0.882	0.791	0.625
g7	0.286	0.082	0.856	0.733	0.856	0.733	0.659	0.435
g8	0.628	0.394	0.984	0.968	0.984	0.968	0.926	0.858
SUM		1.621		6.709		6.709		5.297

The traditional coefficient theta can be calculated by Eq. (2): ρT⁢H=ρT⁢H=_⁢λ⁢i⁢θPC88-1(1-11.621)=0.438. The deflation-corrected theta using *RPC* as the weight factor and the principal component score (θ_*PC*_) as the manifestation of the latent ability (Eq. 24) leads us to an estimate ρT⁢H=_⁢R⁢P⁢C⁢i⁢θPC88-1(1-15.297)=0.927, gamma (Eq. 25) leads to ρ=T⁢H_⁢G⁢i⁢θPC88-1(1-16.709)=0.973, and delta (Eq. 26) to ρα=_⁢D⁢i⁢θPC88-1(1-16.709)=0.973. If the estimates based on *G* or *D* are used as a reference value, the traditional coefficient theta is deflated by 54%, and, if *RPC* is used, 52%. If the raw score (θ*_*X*_*) would be used as a manifestation of the latent ability instead of θ_*PC*_, based on the estimates of correlation in Table 4, the magnitudes of the latter estimates would be ρT⁢H=_⁢R⁢P⁢C⁢i⁢X0.869, ρT⁢H=_⁢G⁢i⁢X0.961, and ρT⁢H=_⁢D⁢i⁢X0.937.

#### Deflation-Corrected Omega and Rho

The traditional coefficients omega and rho use maximum likelihood estimates of factor score (θ_*ML*_) as the manifestation of the latent ability and factor loadings (*λ_*i*_*) as the weighting element in the calculation. Loadings and corresponding statistics related to alternative estimators are collected in Tables 6A,B. As with principal component analysis, because there are no tied pairs between the factor score and items, the estimates by *G* and *D* are identical.

**TABLE 6A:** Factor loadings and related alternative statistics for estimating omega.

Item (*g*)	*λ_*i*_*	(*λ_*i*_*)^2^	1–(*λ_*i*_*)^2^	*D*_*g*θ*ML*_	*(D_*g*_*_θ*ML*_ *)* ^2^	1–*D_*g*θ*ML*_*^2^	*G*_*g*θ*ML*_	*(G_*g*_*_θ*ML*_ *)* ^2^	1–*G*^2^	*RPC*_*g*θ*ML*_	*(RPC_*g*_*_θ*ML*_ *)* ^2^	1–*RPC_*g*θ*ML*_*^2^
g1	0.276	0.076	0.924	0.940	0.884	0.116	0.940	0.884	0.116	0.831	0.691	0.309
g2	0.260	0.068	0.932	0.957	0.916	0.084	0.957	0.916	0.084	0.829	0.688	0.312
g3	0.471	0.222	0.778	0.995	0.990	0.010	0.995	0.990	0.010	0.962	0.926	0.074
g4	0.291	0.085	0.915	0.892	0.796	0.204	0.892	0.796	0.204	0.814	0.663	0.337
g5	0.111	0.012	0.988	0.736	0.542	0.458	0.736	0.542	0.458	0.645	0.415	0.585
g6	0.213	0.045	0.955	0.934	0.872	0.128	0.934	0.872	0.128	0.774	0.599	0.401
g7	0.160	0.026	0.974	0.844	0.712	0.288	0.844	0.712	0.288	0.660	0.435	0.565
g8	0.512	0.262	0.738	0.993	0.986	0.014	0.993	0.986	0.014	0.960	0.922	0.078
SUM	2.294		7.204	7.291		1.302	7.291		1.302	6.475		2.661

**TABLE 6B:** Statistics for calculating rho based on Table 6A.

Item (*g*)	(*λ_*i*_*)^2^/(1–(*λ_*i*_*)^2^)	*(D_*g*_*_θ*ML*_*)*^2^/ (1–*(D_*g*_*_θ*ML*_*)*^2^)	*(G_*g*_*_θ*ML*_*)*^2^/ (1–*(G_*g*_*_θ*ML*_*)*^2^)	*(RPC_*g*_*_θ*ML*_*)*^2^/ (1–*(RPC_*g*_*_θ*ML*_^2^)
g1	0.082	7.591	7.591	2.232
g2	0.073	10.883	10.883	2.202
g3	0.285	99.251	99.251	12.545
g4	0.093	3.894	3.894	1.971
g5	0.012	1.182	1.182	0.711
g6	0.048	6.834	6.834	1.494
g7	0.026	2.476	2.476	0.771
g8	0.355	70.679	70.679	11.776
SUM	0.974	202.791	202.791	33.701

By Eq. (3), the traditional coefficient omega total is calculated as follows: ρω=ρω=_⁢λ⁢i⁢θML(2.294)2(2.294)2+7.204=0.422 and rho by Eq. (4): ρM⁢A⁢X=ρM⁢A⁢X=_⁢λ⁢i⁢θML11+1⁢/⁢0.974=0.493. The deflation-corrected omega using *RPC* as the weight factor (Eq. 27) and the factor score (θ_*ML*_) as the manifestation of the latent ability leads us to an estimate ρω=_⁢R⁢P⁢C⁢i⁢θM⁢L(6.475)2(6.475)2+2.661=0.940 and the corresponding deflation-corrected rho (Eq. 30) is ρM⁢A⁢X=_⁢R⁢P⁢C⁢i⁢θM⁢L11+1⁢/⁢33.701=0.971. Similarly, deflation-corrected omega using gamma (Eq. 28) leads to ρω=_⁢G⁢i⁢θM⁢L(7.291)2(7.291)2+1.302=0.976 and the corresponding deflation-corrected rho (Eq. 31) is ρM⁢A⁢X=_⁢G⁢i⁢θM⁢L11+1⁢/⁢202.791=0.995. Deflation-corrected omega using delta (Eqs. 29) leads to identical estimates in comparison with the estimates by gamma:ρω=_⁢D⁢i⁢θM⁢L(7.291)2(7.291)2+1.302=0.976 and the corresponding deflation-corrected rho (Eq. 32) is ρM⁢A⁢X=_⁢D⁢i⁢θM⁢L11+1⁢/⁢202.791=0.995.

The magnitude of the estimates based on the form of maximal reliability and *G* and *D* as the weighting factor (0.995), feel intuitively overestimates. This is reasoned by the fact that the formula of maximal reliability is sensitive for high values of loadings. With very high values of loading—as here *G* = *D* = 0.995 for item *g*3 referring to a fact that after the test takers are ordered by the factor score variable, 99.5% of the test takers are in the same order in both item and score—the statistic $\lambda_{i}^{2}\,/\,\left(1-\lambda_{i}^{2}\right)$ may give an artificially high value leading to artificially high estimate of reliability. However, if the estimates based on *G* or *D* are used as a reference value, the traditional coefficient omega and rho are deflated by 57 and 50%, and, if *RPC* is used, 55 and 49%, respectively. If the raw score (*X*) would be used as a manifestation of the latent ability instead of θ_*ML*_, the magnitudes of the DCERs based on omega would be ρω=_⁢R⁢P⁢C⁢i⁢X0.895, ρω=_⁢G⁢i⁢X0.967, and ρω=_⁢D⁢i⁢X0.947 and DCERs based on rho ρM⁢A⁢X=_⁢R⁢P⁢C⁢i⁢X0.929, ρM⁢A⁢X=_⁢G⁢i⁢X0.979, and ρω=_⁢D⁢i⁢X0.961.

The estimates of reliability above are summarized in Table 7. Different interpretations of the varying estimators are discussed in the next section. Anyhow, just by comparing the overall level of magnitudes of the traditional estimates and the estimates by different DCERs we may conclude that all the DCERs seem to refer to a reliability which is notably higher than the ones indicated by the traditional estimators. If one uses the raw scores, instead of ρ_α_ = 0.245, the true reliability seems to be around 0.914 (on average), varying between 0.790 and 0.979 depending on which form is used as the base and which deflation-corrected estimator of correlation is used as the weighting element. Knowing the interpretation of *RPC*, *G* and *D*, the high magnitude of reliability by DCERs refer to the fact that the score is highly capable of ordering the test takers in a logical order by their latent ability. Of the estimators, the ones based on coefficient alpha are the most conservative and the ones based on rho the most liberal. In this case, the estimators of correlation based on probability (*G* and *D*) tend to lead somewhat higher estimates than the one based on covariance (*RPC*). This is not a general characteristic though.

**TABLE 7:** Summary of estimates of reliability.

		Traditional estimate	DCERs with the traditional score			DCERs with the raw score		
Form	Score type (θ)	*R*	*D*	*G*	*RPC*	*D*	*G*	*RPC*
Alfa	Raw score (θ_*X*_)	0.245	0.856	0.885	0.790	0.856	0.885	0.790
Theta	Principal component score (θ_*PC*_)	0.444	0.973	0.973	0.927	0.937	0.961	0.869
Omega	Factor score (θ_*ML*_)	0.422	0.976	0.976	0.940	0.947	0.967	0.895
Rho	Factor score (θ_*ML*_)	0.493	0.995	0.995	0.971	0.961	0.979	0.929

### Different Interpretation of Different Estimators of Reliability

The article did not tackle the issue of differences between the estimators of correlation. Notably, PMC, *RPC*, and *G* (as well as *D*) discussed in the article indicate different aspects of the correlation: PMC estimates the *observed correlation* between two variables, and this is radically deflated in the measurement modeling settings. *RPC* estimates the *inferred correlation* of two unobservable continuous variables by their ordinal manifestations. *G* and *D* estimate the *probability* that the test takers are in the same order both in an item and a score. The outcome of different estimators of reliability may, then, indicate different viewpoints of reliability.

Chalmers (2017) is skeptical of the usefulness of coefficients using *RPC* in practical settings because *RPC* refers to correlation between unobservable and unreachable variables and, therefore, the outcome may be useless in the factual interpretation of the observed score. He proposes that using *RPC* leads to infer something about *theoretical reliability*. However, some estimators of reliability such as ordinal alpha and theta by Zumbo et al. (2007; see also Gadermann et al., 2012), factually, use *RPC* in the estimation. Comparing the estimators related to *RPC* in Eqs. (21), (24), and (27) and (39) to (43) with ordinal alpha or ordinal theta based on the matrix of inter-item *RPCs* instead of matrix of PMCs may be worth studying.

Estimators based on *G* and *D* refer to observed variables and, therefore, the outcome may be more useful than those by *RPC* in the factual analysis of the observed score. Knowing the interpretation of *G* and *D* in the measurement settings (see Metsämuuronen, 2021a,b), estimators (22) and (23), (25) and (26), (31) and (32), and (44) to (48) reflect the average proportion of logically ordered test takers in all items as a whole. In this, the estimators based on *D* are more conservative than the ones based on *G*.

A relevant question is, how different is the interpretation of the estimates by *G* (or *D*) in comparison to those by PMC or *RPC*? Knowing that *G* estimates the probability that the test takers are in the same order in the item and in the score, the ultimate magnitude of reliability by the estimators based on *G* would indicate that *all* items discriminate the higher-performing test takers from the lower-performing test takers in a deterministic manner after the test takers are ordered by the score. The same interpretation would be obtained when using *RPC* except that *RPC* can reach the value *RPC* = 1 only approximatively. From this viewpoint, the deflation-corrected estimators in Eqs. (24) to (32) related to *RPC*, *G*, and *D* seems to refer strictly to the *discrimination power* of the score. This makes sense from the standard error of measurement viewpoint. Notably, under the condition of deterministic item discrimination, the estimators using PMC cannot reach the perfect reliability because the estimates by PMC cannot detect the deterministic correlation unless the number of categories is equal in the variables. More studies and theoretical work in the interpretation of the estimators would enrich us.

Some typological characteristics of different estimators of the estimators described in the article are summarized in Table 8. Notably, again, *RPC*, *G*, and *D* are not the only options for DCERs; further studies related to such estimators as r-bireg- and r-polyreg correlations, *G*_2_, *D*_2_, as well as attenuation-corrected *Rit* and *eta*, as examples, would be beneficial (see footnote 6).

**TABLE 8:** General typological characteristics of selected options of DCERs.

		Weight *w*_*i*_	
		*RPC*	*G* and *D*
Base	General characteristics	• Reflects *latent* reliability; not strictly related to the observed score nor observed items • Leads to theoretical interpretation of reliability • Based on covariance • Suitable for binary and polytomous items • Not simple to calculate	• Reflects reliability of the *observed* score • Leads to practical interpretation of reliability • Based on probability • *D* is more conservative than *G* • Suitable for binary items and polytomous items with < 4 categories (*D*) or with < 5 categories (*G*) • Simple to calculate even manually

Alpha	• Always underestimates population reliability • Very conservative nature • Gives estimates even with small sample sizes • Reaches the perfect reliability (*REL* = 1) when *w*_*i*_ = 1, and *σ_*i*_* = *σ_*j*_*	$\frac{k}{k-1}\,\left(1-\frac{\sum_{i=1}^{k}\sigma_{i}^{2}}{{\left(\sum_{i=1}^{k}\sigma_{i}\times R\,P\,C_{i\,\theta }\right)}^{2}}\right)$	$\frac{k}{k-1}\,\left(1-\frac{\sum_{i=1}^{k}\sigma_{i}^{2}}{{\left(\sum_{i=1}^{k}\sigma_{i}\times G_{i\,\theta }\right)}^{2}}\right)$

Theta	• Maximizes alpha • Conservative nature • Gives estimates even with small sample sizes • Reaches the perfect reliability (*REL* = 1) when *w*_*i*_ = 1	$\frac{k}{k-1}\,\left(1-\frac{1}{\sum_{i=1}^{k}R\,P\,C_{i\,\theta }^{2}}\right)$	$\frac{k}{k-1}\,\left(1-\frac{1}{\sum_{i=1}^{k}G_{i\,\theta }^{2}}\right)$

Omega	• Estimates always higher than alpha • Least conservative nature • Gives estimates even with small sample sizes • Reaches the perfect reliability (*REL* = 1) when *w*_*i*_ = 1	$\frac{{\left(\sum_{i=1}^{k}R\,P\,C_{i\,\theta }\right)}^{2}}{{\left(\sum_{i=1}^{k}R\,P\,C_{i\,\theta }\right)}^{2}+\sum_{g=1}^{k}\left(1-R\,P\,C_{i\,\theta }^{2}\right)}$	$\frac{{\left(\sum_{i=1}^{k}G_{i\,\theta }\right)}^{2}}{{\left(\sum_{i=1}^{k}G_{i\,\theta }\right)}^{2}+\sum_{g=1}^{k}\left(1-G_{i\,\theta }^{2}\right)}$

Rho (maximal reliability)	• Maximizes omega • Liberal nature; may overestimate reliability with small sample sizes • Cannot be calculated if deterministic patterns (λ = 1) even in one item • Cannot reach the perfect reliability (*REL* = 1) • Not the best option for small samples	$\frac{1}{1+\frac{1}{\sum_{i=1}^{k}\left(R\,P\,C_{i\,\theta }^{2}\,/\,\left(1-R\,P\,C_{i\,\theta }^{2}\right)\right)}}$	$\frac{1}{1+\frac{1}{\sum_{i=1}^{k}\left(G_{i\,\theta }^{2}\,/\,\left(1-G_{i\,\theta }^{2}\right)\right)}}$

### Known Limitations of the Treatment

The empirical section offers, obviously, just examples of what kind of effect would be obtained if an estimator with smaller quantity of deflation is used as the linking factor between the latent variables and the item. Wider comparisons of different estimators would benefit us to select most suitable estimators of correlation as the linking factors for different variables, estimators of reliability and different type of datasets. Systematic simulations also in this area would enrich us.

The DCERs in the article were given just as examples—their characteristics were not studied in-depth. Specifically, the estimators based on omega and rho are, by far, theoretical options in the settings related to factor analysis and structural equation modeling because they may require new procedures where the *outcome* of factor loadings would be (essentially) *RPC* or *G* instead of (essentially) PMC. Notably, the current procedures of using *RPC* in EFA and SEM may *start* by using *RPC* in forming the correlation matrix, but the outcome of the loadings seems to be still, essentially, PMC. Also, Chalmers (2017) critique against the use of *RPC* in estimating reliability is worth noting. More studies in this regard would benefit us.

The study did not tackle the question of possible overestimation of reliability when using deflation-corrected estimators of reliability. *Assuming* that *RPC* does not overestimate the true correlation, it may be relevant to conclude that a deflation-corrected estimator based on *RPC* such as Eqs. (21), (24), (27), and (30) would not overestimate reliability. What would be the mechanism for overestimation? It may be possible that the estimators based on rho overestimate the reliability in the real-world settings; this would be a reasonable consequence of the results by Aquirre-Urreta et al. (2019) that rho may overestimate the true reliability with finite samples familiar in real-world testing settings with small or smallish number of test takers. From this viewpoint, the estimators based on alpha, theta and omega seem to give more conservative estimates. Theoretical and empirical studies in the area would be beneficial.

Finally, in several places in the article a loose wording concerning the deflation in the estimates of reliability was described as “remarkable” or “notable.” Based on the behavior of PMC, it is expected that the effect of changing PMC with better behaving estimators of correlation in the estimators of reliability is “remarkable” or maybe even “dramatical” when the test is very easy or very demanding to the target group or with tests with incremental difficulty levels as are usual in the educational testing settings; PMC is severely deflated in these cases. Also, with the tests of incremental difficulty level where part of the test items may be very easy and part may be very demanding as is usual in the achievement testing, we may expect remarkable difference between the traditional estimators and deflation-corrected ones. However, when all items are of medium difficulty level, the effect may not be as notable. Wider empirical studies and simulations would enrich us in this regard.

## Data Availability Statement

The datasets presented in this study can be found in online repositories. The names of the repository/repositories and accession number(s) can be found in the article/Supplementary Material.

## Ethics Statement

Ethical review and approval was not required for the study on human participants in accordance with the local legislation and institutional requirements. Written informed consent from the participants’ legal guardian/next of kin was not required to participate in this study in accordance with the national legislation and the institutional requirements.

## Author Contributions

JM contributed alone in the article.

## Conflict of Interest

The author declares that the research was conducted in the absence of any commercial or financial relationships that could be construed as a potential conflict of interest.

## Publisher’s Note

All claims expressed in this article are solely those of the authors and do not necessarily represent those of their affiliated organizations, or those of the publisher, the editors and the reviewers. Any product that may be evaluated in this article, or claim that may be made by its manufacturer, is not guaranteed or endorsed by the publisher.

## Footnotes

1 The basic contents of the derivation of underestimation of PMC in the measurement modeling settings, later elaborated in Metsämuuronen (2016), were initially published in Metsämuuronen (2009); in Finnish.

2 The value depends on, to some extent, the number of bins in the variable with wider scale. For example, with 10, 20, 30, 200, and 1,000 bins, the maximum value is 0.8704, 0.8671, 0.8665, 0.8660, and 0.8660, respectively. This is easy to confirm by forming these sets of variables.

3 We recall that, although the traditional formula of ρ_*BS*_ is usually expressed by using PMC between two parallel tests, it can be expressed also by using $\sigma_{X}^{2}$ in the form familiar from ρ_*FR*_ (see Lord et al., 1968).

4 The effectiveness is expected because, in their original context, *ρ*_*TH*_ maximizes *ρ*_α_ (Greene and Carmines, 1980), the magnitude of the estimates by *ρ*_*MAX*_is higher than those by *ρ*_ω_ (Cheng et al., 2012), and all three give higher value than alpha if the item–score correlations or loadings are not equal (e.g., Cheng et al., 2012).

5 Notably, *RPC* cannot reach the perfect 1. With enhanced procedures of the estimation by adding a very small number like 10^–50^ to each element of logarithm and when the embedded PMC ≈ 1 such as 0.99999999, *RPC* ≈ 1.

6 The dataset used in this article is a simple one intending to lead the reader to the concepts and relevant estimators by offering all necessary calculations in Supplementary Appendix 1. A dataset comprising a more in-depth comparison of different estimators is also available at http://dx.doi.org/10.13140/RG.2.2.27971.94241. This wider dataset is a simulation including 1,440 estimates of reliability drawn from the same real-life dataset as used in Supplementary Appendix 1, however, so that the sample size is varied (*n* = 25, 50, 100, 200) as well as the number of categories and difficulty levels in the items and the score, and more options for the weight element are compared: traditional weights, *RPC*, *G*, *D*, *RREG*, *G*_2_, *D*_2_, *RAC*, and *EAC*. Unlike the dataset used in this article, the score variables in the larger dataset do not include θ_*IRT*_ and θ_*PI*_ though.
