# Supplementary material for: Deflation-Corrected Estimators of Reliability
Source: Front Psychol. 2022 Jan 4;12:748672. doi: 10.3389/fpsyg.2021.748672 (PMC8781775; doi:10.3389/fpsyg.2021.748672)
Supplement: Supplementary file 1 [file Data_Sheet_1.docx]

| Appendix 1. Dataset used in the example and the related statistics and calculation of the estimates | | | | | | | | | | | | | | | | | | | | | | | | | | | | | | | | | | |  |
| --- | --- | --- | --- | --- | --- | --- | --- | --- | --- | --- | --- | --- | --- | --- | --- | --- | --- | --- | --- | --- | --- | --- | --- | --- | --- | --- | --- | --- | --- | --- | --- | --- | --- | --- | --- |
| **Dataset and score variables** | | | | | | | | | | | | | | | | | | | | | | | | | | | | | | | | | | | |
| V1 | V2 | V3 | V4 | V5 | V6 | V7 | V8 | V9 | V10 | V11 | V12 | V13 | V14 | V15 | V16 | V17 | V18 | V19 | V20 | V21 | V22 | V23 | V24 | V25 | V26 | V27 | V28 | V29 | V30 | X | X/pi | IRT | FA | PC | |
| 1 | 0 | 0 | 0 | 1 | 1 | 0 | 0 | 1 | 0 | 0 | 0 | 1 | 0 | 0 | 0 | 1 | 0 | 0 | 0 | 0 | 0 | 1 | 1 | 0 | 0 | 1 | 0 | 0 | 0 | 9 | 12.858 | -0.989 | -2.177 | -2.339 | |
| 1 | 1 | 0 | 0 | 0 | 1 | 1 | 1 | 0 | 0 | 1 | 0 | 0 | 0 | 1 | 0 | 0 | 1 | 0 | 0 | 0 | 0 | 0 | 0 | 1 | 1 | 0 | 0 | 0 | 0 | 10 | 12.993 | -0.818 | -1.680 | -1.774 | |
| 0 | 1 | 0 | 1 | 1 | 1 | 0 | 0 | 0 | 0 | 1 | 0 | 0 | 1 | 0 | 1 | 0 | 1 | 0 | 1 | 0 | 0 | 0 | 1 | 0 | 0 | 0 | 1 | 0 | 0 | 11 | 15.213 | -0.654 | -1.864 | -1.844 | |
| 1 | 0 | 0 | 1 | 1 | 1 | 0 | 0 | 1 | 1 | 1 | 0 | 0 | 0 | 0 | 0 | 0 | 0 | 0 | 0 | 1 | 1 | 1 | 0 | 1 | 0 | 0 | 0 | 0 | 0 | 11 | 15.252 | -0.654 | -1.850 | -1.895 | |
| 1 | 1 | 1 | 1 | 1 | 0 | 1 | 1 | 0 | 1 | 1 | 0 | 0 | 0 | 1 | 0 | 1 | 0 | 0 | 1 | 0 | 0 | 0 | 0 | 0 | 0 | 1 | 0 | 0 | 0 | 13 | 17.428 | -0.339 | -1.291 | -1.329 | |
| 1 | 1 | 0 | 0 | 1 | 1 | 1 | 0 | 1 | 1 | 1 | 0 | 0 | 0 | 0 | 1 | 1 | 1 | 0 | 0 | 0 | 0 | 0 | 0 | 0 | 1 | 1 | 0 | 0 | 0 | 13 | 17.590 | -0.339 | -1.779 | -1.728 | |
| 0 | 0 | 1 | 0 | 0 | 0 | 1 | 0 | 0 | 1 | 0 | 1 | 1 | 1 | 1 | 0 | 1 | 1 | 1 | 1 | 0 | 0 | 0 | 1 | 0 | 0 | 1 | 0 | 0 | 0 | 13 | 18.502 | -0.339 | -0.751 | -1.079 | |
| 1 | 1 | 1 | 1 | 1 | 1 | 0 | 1 | 0 | 0 | 0 | 0 | 0 | 0 | 1 | 0 | 0 | 1 | 0 | 0 | 1 | 0 | 0 | 1 | 0 | 0 | 1 | 0 | 1 | 0 | 13 | 18.901 | -0.339 | -1.366 | -1.377 | |
| 1 | 1 | 1 | 1 | 1 | 1 | 1 | 1 | 0 | 0 | 0 | 1 | 0 | 1 | 1 | 0 | 0 | 1 | 1 | 1 | 0 | 0 | 0 | 0 | 1 | 1 | 0 | 0 | 0 | 0 | 16 | 21.110 | 0.122 | -0.395 | -0.573 | |
| 1 | 1 | 1 | 1 | 1 | 1 | 0 | 0 | 1 | 0 | 1 | 1 | 0 | 1 | 0 | 0 | 1 | 0 | 0 | 1 | 1 | 1 | 0 | 1 | 0 | 1 | 0 | 0 | 0 | 0 | 16 | 21.386 | 0.122 | -0.948 | -0.962 | |
| 1 | 1 | 0 | 1 | 1 | 1 | 1 | 1 | 0 | 1 | 1 | 1 | 0 | 0 | 1 | 1 | 0 | 0 | 1 | 0 | 0 | 0 | 0 | 1 | 0 | 1 | 1 | 0 | 0 | 0 | 16 | 21.800 | 0.122 | -0.878 | -0.969 | |
| 1 | 1 | 0 | 1 | 0 | 0 | 1 | 0 | 1 | 1 | 1 | 1 | 0 | 0 | 0 | 1 | 1 | 1 | 0 | 0 | 0 | 1 | 0 | 0 | 0 | 1 | 1 | 0 | 1 | 0 | 15 | 22.079 | -0.032 | -1.112 | -1.067 | |
| 1 | 1 | 0 | 0 | 1 | 1 | 1 | 0 | 1 | 1 | 1 | 1 | 1 | 0 | 1 | 1 | 1 | 0 | 1 | 1 | 0 | 1 | 1 | 0 | 0 | 0 | 0 | 0 | 0 | 0 | 17 | 23.025 | 0.278 | -0.621 | -0.760 | |
| 1 | 1 | 0 | 0 | 1 | 1 | 1 | 1 | 1 | 1 | 0 | 1 | 1 | 1 | 0 | 1 | 1 | 1 | 1 | 1 | 1 | 1 | 0 | 0 | 0 | 0 | 0 | 0 | 0 | 0 | 18 | 24.243 | 0.437 | -0.250 | -0.402 | |
| 1 | 1 | 0 | 1 | 1 | 1 | 1 | 1 | 1 | 0 | 1 | 1 | 1 | 1 | 1 | 0 | 1 | 1 | 1 | 1 | 0 | 0 | 0 | 0 | 1 | 1 | 0 | 0 | 0 | 0 | 19 | 25.241 | 0.600 | -0.193 | -0.291 | |
| 1 | 1 | 1 | 0 | 1 | 1 | 1 | 1 | 1 | 1 | 1 | 1 | 1 | 0 | 1 | 1 | 0 | 1 | 0 | 1 | 1 | 0 | 1 | 1 | 0 | 0 | 0 | 0 | 0 | 0 | 19 | 25.367 | 0.600 | -0.205 | -0.245 | |
| 0 | 1 | 1 | 1 | 1 | 1 | 1 | 1 | 1 | 1 | 1 | 1 | 1 | 0 | 0 | 0 | 1 | 1 | 1 | 0 | 0 | 1 | 0 | 0 | 1 | 1 | 0 | 1 | 0 | 0 | 19 | 25.894 | 0.600 | 0.003 | -0.164 | |
| 1 | 0 | 1 | 1 | 0 | 1 | 1 | 1 | 1 | 1 | 1 | 1 | 0 | 1 | 1 | 1 | 1 | 1 | 1 | 0 | 0 | 1 | 0 | 0 | 0 | 1 | 0 | 1 | 0 | 0 | 19 | 26.065 | 0.600 | -0.220 | -0.332 | |
| 0 | 1 | 1 | 1 | 0 | 1 | 1 | 1 | 1 | 0 | 1 | 0 | 0 | 1 | 1 | 0 | 1 | 1 | 0 | 1 | 1 | 0 | 1 | 1 | 0 | 0 | 0 | 1 | 1 | 0 | 18 | 26.168 | 0.437 | -0.558 | -0.457 | |
| 1 | 1 | 1 | 1 | 1 | 1 | 1 | 1 | 1 | 1 | 0 | 0 | 1 | 1 | 0 | 0 | 0 | 1 | 0 | 0 | 0 | 0 | 1 | 1 | 1 | 1 | 1 | 1 | 0 | 0 | 19 | 26.784 | 0.600 | -0.413 | -0.366 | |
| 1 | 1 | 1 | 1 | 0 | 1 | 0 | 0 | 1 | 1 | 0 | 1 | 0 | 1 | 0 | 1 | 1 | 1 | 0 | 0 | 1 | 1 | 0 | 1 | 0 | 1 | 0 | 1 | 0 | 1 | 18 | 27.773 | 0.437 | -0.629 | -0.557 | |
| 1 | 1 | 1 | 1 | 1 | 1 | 1 | 1 | 0 | 1 | 1 | 1 | 1 | 0 | 0 | 0 | 1 | 1 | 1 | 1 | 1 | 0 | 1 | 1 | 1 | 0 | 0 | 1 | 0 | 0 | 21 | 28.894 | 0.947 | 0.232 | 0.142 | |
| 0 | 1 | 1 | 1 | 1 | 0 | 1 | 1 | 1 | 1 | 1 | 1 | 0 | 1 | 1 | 1 | 1 | 1 | 1 | 1 | 0 | 0 | 1 | 1 | 1 | 0 | 0 | 1 | 0 | 0 | 21 | 29.361 | 0.947 | 0.169 | 0.176 | |
| 1 | 1 | 1 | 1 | 1 | 1 | 1 | 1 | 1 | 0 | 1 | 1 | 1 | 0 | 1 | 0 | 0 | 1 | 1 | 0 | 1 | 1 | 1 | 0 | 0 | 1 | 0 | 1 | 1 | 0 | 21 | 29.951 | 0.947 | 0.284 | 0.200 | |
| 1 | 1 | 1 | 1 | 1 | 1 | 1 | 1 | 1 | 0 | 1 | 1 | 1 | 1 | 1 | 1 | 1 | 0 | 1 | 0 | 1 | 1 | 0 | 1 | 1 | 1 | 0 | 0 | 0 | 0 | 22 | 30.056 | 1.135 | 0.320 | 0.230 | |
| 1 | 1 | 1 | 1 | 1 | 1 | 1 | 1 | 1 | 1 | 1 | 1 | 1 | 1 | 1 | 0 | 1 | 1 | 1 | 1 | 0 | 1 | 0 | 1 | 1 | 1 | 0 | 0 | 0 | 0 | 23 | 30.834 | 1.337 | 0.478 | 0.426 | |
| 1 | 1 | 1 | 0 | 1 | 1 | 1 | 1 | 1 | 1 | 1 | 1 | 1 | 1 | 1 | 1 | 1 | 1 | 0 | 1 | 0 | 0 | 1 | 0 | 0 | 1 | 1 | 0 | 1 | 0 | 22 | 31.652 | 1.135 | -0.065 | -0.027 | |
| 1 | 1 | 1 | 1 | 1 | 1 | 1 | 1 | 0 | 0 | 1 | 0 | 1 | 0 | 1 | 1 | 1 | 1 | 0 | 1 | 1 | 1 | 1 | 1 | 0 | 0 | 0 | 0 | 1 | 1 | 21 | 32.567 | 0.947 | -0.125 | -0.004 | |
| 1 | 1 | 1 | 1 | 0 | 1 | 1 | 0 | 1 | 1 | 1 | 1 | 1 | 1 | 0 | 1 | 1 | 1 | 1 | 0 | 1 | 0 | 1 | 1 | 1 | 1 | 1 | 1 | 0 | 0 | 23 | 33.015 | 1.337 | 0.233 | 0.238 | |
| 1 | 1 | 1 | 1 | 1 | 1 | 1 | 1 | 0 | 1 | 0 | 1 | 1 | 1 | 1 | 1 | 0 | 1 | 1 | 1 | 0 | 1 | 0 | 0 | 1 | 1 | 0 | 1 | 0 | 1 | 22 | 33.285 | 1.135 | 0.448 | 0.365 | |
|  |  |  |  |  |  |  |  |  |  |  |  |  |  |  |  |  |  |  |  |  |  |  |  |  |  |  |  |  |  |  |  |  |  |  | |
|  |  |  |  |  |  |  |  |  |  |  |  |  |  |  |  |  |  |  |  |  |  |  |  |  |  |  |  |  |  |  |  |  |  |  | |
|  |  |  |  |  |  |  |  |  |  |  |  |  |  |  |  |  |  |  |  |  |  |  |  |  |  |  |  |  |  |  |  |  |  |  | |
| V1 | V2 | V3 | V4 | V5 | V6 | V7 | V8 | V9 | V10 | V11 | V12 | V13 | V14 | V15 | V16 | V17 | V18 | V19 | V20 | V21 | V22 | V23 | V24 | V25 | V26 | V27 | V28 | V29 | V30 | X | X/pi | IRT | FA | PC | |
| 1 | 1 | 1 | 1 | 1 | 1 | 1 | 1 | 1 | 1 | 0 | 1 | 1 | 1 | 1 | 0 | 1 | 1 | 1 | 1 | 1 | 1 | 1 | 1 | 1 | 0 | 0 | 1 | 0 | 0 | 24 | 33.316 | 1.559 | 0.726 | 0.688 | |
| 1 | 1 | 1 | 1 | 1 | 1 | 1 | 1 | 1 | 1 | 1 | 1 | 1 | 1 | 1 | 1 | 1 | 1 | 1 | 0 | 1 | 1 | 1 | 1 | 1 | 0 | 0 | 1 | 0 | 0 | 25 | 34.749 | 1.806 | 0.732 | 0.737 | |
| 1 | 1 | 1 | 1 | 1 | 0 | 1 | 1 | 1 | 1 | 1 | 1 | 1 | 1 | 1 | 1 | 0 | 1 | 1 | 1 | 1 | 1 | 1 | 1 | 0 | 1 | 1 | 1 | 0 | 0 | 25 | 35.616 | 1.806 | 0.757 | 0.794 | |
| 1 | 1 | 1 | 1 | 1 | 1 | 1 | 1 | 1 | 1 | 1 | 1 | 1 | 1 | 1 | 1 | 1 | 1 | 1 | 1 | 1 | 1 | 1 | 1 | 0 | 1 | 0 | 1 | 0 | 0 | 26 | 35.994 | 2.090 | 0.801 | 0.847 | |
| 1 | 1 | 1 | 1 | 1 | 1 | 1 | 1 | 1 | 1 | 1 | 1 | 1 | 1 | 1 | 0 | 1 | 1 | 1 | 1 | 1 | 1 | 0 | 1 | 1 | 1 | 1 | 1 | 0 | 0 | 26 | 36.620 | 2.090 | 0.761 | 0.778 | |
| 1 | 1 | 1 | 1 | 0 | 1 | 1 | 1 | 1 | 1 | 1 | 1 | 1 | 1 | 1 | 1 | 1 | 1 | 1 | 1 | 1 | 1 | 1 | 1 | 1 | 1 | 1 | 1 | 0 | 0 | 27 | 38.763 | 2.430 | 0.902 | 0.954 | |
| 1 | 1 | 1 | 1 | 1 | 1 | 1 | 1 | 1 | 1 | 1 | 1 | 1 | 1 | 1 | 1 | 0 | 1 | 1 | 1 | 1 | 1 | 1 | 1 | 1 | 1 | 1 | 0 | 1 | 0 | 27 | 39.343 | 2.430 | 0.890 | 0.957 | |
| 1 | 1 | 1 | 1 | 1 | 1 | 1 | 1 | 1 | 1 | 1 | 1 | 1 | 1 | 1 | 1 | 1 | 1 | 1 | 1 | 1 | 1 | 1 | 1 | 1 | 0 | 0 | 0 | 1 | 1 | 27 | 41.209 | 2.430 | 0.954 | 1.040 | |
| 1 | 1 | 1 | 1 | 1 | 1 | 1 | 1 | 1 | 1 | 1 | 1 | 1 | 1 | 1 | 1 | 0 | 1 | 1 | 1 | 1 | 1 | 1 | 1 | 1 | 1 | 1 | 1 | 1 | 0 | 28 | 41.343 | 2.861 | 0.994 | 1.090 | |
| 1 | 1 | 1 | 1 | 1 | 1 | 1 | 1 | 1 | 0 | 1 | 1 | 1 | 1 | 1 | 1 | 1 | 1 | 1 | 1 | 1 | 1 | 1 | 1 | 1 | 1 | 1 | 1 | 1 | 0 | 28 | 41.380 | 2.861 | 0.951 | 1.043 | |
| 1 | 1 | 1 | 1 | 1 | 0 | 1 | 1 | 1 | 1 | 1 | 1 | 1 | 1 | 1 | 1 | 1 | 1 | 1 | 1 | 1 | 1 | 1 | 1 | 1 | 1 | 0 | 0 | 1 | 1 | 27 | 41.561 | 2.430 | 1.045 | 1.161 | |
| 1 | 1 | 1 | 1 | 1 | 1 | 1 | 1 | 0 | 1 | 1 | 1 | 1 | 1 | 1 | 0 | 1 | 1 | 1 | 1 | 1 | 1 | 1 | 1 | 1 | 1 | 0 | 1 | 1 | 1 | 27 | 41.776 | 2.430 | 0.988 | 1.062 | |
| 1 | 1 | 1 | 1 | 1 | 1 | 1 | 1 | 1 | 1 | 0 | 1 | 1 | 1 | 0 | 1 | 1 | 1 | 1 | 1 | 1 | 1 | 1 | 1 | 0 | 1 | 1 | 1 | 1 | 1 | 27 | 42.441 | 2.430 | 0.837 | 0.930 | |
| 1 | 1 | 1 | 1 | 1 | 1 | 1 | 1 | 1 | 1 | 1 | 1 | 1 | 0 | 1 | 1 | 1 | 1 | 0 | 0 | 1 | 1 | 1 | 1 | 1 | 1 | 1 | 1 | 1 | 1 | 27 | 42.442 | 2.430 | 0.631 | 0.820 | |
| 1 | 1 | 1 | 1 | 1 | 1 | 1 | 1 | 1 | 1 | 1 | 1 | 1 | 1 | 1 | 1 | 1 | 1 | 1 | 1 | 1 | 1 | 1 | 1 | 1 | 1 | 1 | 1 | 1 | 0 | 29 | 42.731 | 3.475 | 1.056 | 1.178 | |
| 1 | 1 | 1 | 1 | 1 | 0 | 1 | 1 | 1 | 1 | 1 | 1 | 1 | 1 | 1 | 1 | 1 | 1 | 1 | 1 | 1 | 1 | 1 | 1 | 1 | 1 | 1 | 1 | 0 | 1 | 28 | 42.958 | 2.861 | 1.079 | 1.200 | |
| 1 | 1 | 1 | 1 | 1 | 1 | 1 | 1 | 1 | 1 | 1 | 1 | 1 | 0 | 1 | 1 | 1 | 1 | 1 | 1 | 1 | 1 | 1 | 1 | 1 | 1 | 1 | 0 | 1 | 1 | 28 | 43.428 | 2.861 | 0.927 | 1.044 | |
| 1 | 1 | 1 | 1 | 1 | 1 | 1 | 1 | 1 | 1 | 1 | 1 | 1 | 1 | 1 | 1 | 1 | 1 | 1 | 1 | 1 | 1 | 1 | 1 | 1 | 1 | 1 | 0 | 1 | 1 | 29 | 44.898 | 3.475 | 1.068 | 1.204 | |
| 1 | 1 | 1 | 1 | 1 | 1 | 1 | 1 | 1 | 1 | 1 | 1 | 1 | 1 | 1 | 0 | 1 | 1 | 1 | 1 | 1 | 1 | 1 | 1 | 1 | 1 | 1 | 1 | 1 | 1 | 29 | 45.231 | 3.475 | 1.101 | 1.232 | |

*X* = unweighted sum, *X*/*pi* = sum weighted by 1/p_i_, IRT = sum weighted by IRT modeling, FA_ML = sum weighted by Factor analysis with ML-estimation, PC = sum weighted by principal component analysis

**Statistics related to the estimates of reliability**

|  | V1 | V2 | V3 | V4 | V5 | V6 | V7 | V8 | V9 | V10 | V11 | V12 | V13 | V14 | V15 |
| --- | --- | --- | --- | --- | --- | --- | --- | --- | --- | --- | --- | --- | --- | --- | --- |
| *p* | 0.898 | 0.918 | 0.796 | 0.837 | 0.837 | 0.857 | 0.878 | 0.796 | 0.776 | 0.755 | 0.796 | 0.796 | 0.714 | 0.673 | 0.735 |
| $\sigma_{i}^{2}=p\left(1-p\right)$ | 0.092 | 0.075 | 0.162 | 0.137 | 0.137 | 0.122 | 0.107 | 0.162 | 0.174 | 0.185 | 0.162 | 0.162 | 0.204 | 0.220 | 0.195 |
| *B* (IRT) | -1.441 | -1.707 | -0.520 | -0.837 | -0.837 | -1.017 | -1.215 | -0.520 | -0.377 | -0.240 | -0.520 | -0.520 | 0.016 | 0.256 | -0.110 |
| $\lambda_{i\text{θ}ML}$ | 0.204 | 0.376 | 0.635 | 0.420 | 0.213 | 0.006 | 0.556 | 0.588 | 0.330 | 0.372 | 0.203 | 0.670 | 0.657 | 0.484 | 0.454 |
| $\lambda_{i\text{θPC}}$ | 0.229 | 0.425 | 0.669 | 0.466 | 0.227 | 0.008 | 0.565 | 0.614 | 0.365 | 0.391 | 0.241 | 0.671 | 0.67 | 0.503 | 0.468 |
| Somers D*_iX_* | 0.523 | 0.778 | 0.854 | 0.671 | 0.396 | 0.092 | 0.849 | 0.777 | 0.550 | 0.507 | 0.405 | 0.836 | 0.845 | 0.564 | 0.558 |
| *σ_i_* ×*R_iX_* | 0.082 | 0.114 | 0.253 | 0.168 | 0.090 | 0.019 | 0.171 | 0.228 | 0.172 | 0.172 | 0.112 | 0.256 | 0.300 | 0.226 | 0.190 |
| *σ_i_* ×*RPC_iX_* | 0.139 | 0.203 | 0.351 | 0.253 | 0.140 | 0.029 | 0.270 | 0.308 | 0.226 | 0.229 | 0.172 | 0.346 | 0.354 | 0.278 | 0.258 |
| *σ_i_* ×*G_iX_* | 0.168 | 0.223 | 0.354 | 0.257 | 0.154 | 0.034 | 0.286 | 0.320 | 0.240 | 0.229 | 0.172 | 0.347 | 0.391 | 0.277 | 0.259 |
| *σ_i_* ×*D_iX_* | 0.158 | 0.213 | 0.344 | 0.248 | 0.146 | 0.032 | 0.278 | 0.313 | 0.229 | 0.218 | 0.163 | 0.337 | 0.382 | 0.264 | 0.246 |
| *R_iX_* | 0.272 | 0.416 | 0.627 | 0.454 | 0.244 | 0.055 | 0.521 | 0.566 | 0.412 | 0.400 | 0.277 | 0.636 | 0.663 | 0.482 | 0.430 |
| *R_iX/Pi_* | 0.266 | 0.389 | 0.615 | 0.454 | 0.224 | 0.034 | 0.472 | 0.526 | 0.376 | 0.391 | 0.243 | 0.585 | 0.639 | 0.447 | 0.405 |
| *R_iIRT_* | 0.272 | 0.372 | 0.575 | 0.430 | 0.247 | 0.049 | 0.467 | 0.525 | 0.394 | 0.384 | 0.283 | 0.579 | 0.627 | 0.455 | 0.423 |
| *R_iML_* | 0.216 | 0.398 | 0.671 | 0.444 | 0.225 | 0.006 | 0.588 | 0.622 | 0.349 | 0.393 | 0.215 | 0.709 | 0.695 | 0.512 | 0.480 |
| *R_iPC_* | 0.229 | 0.425 | 0.669 | 0.466 | 0.227 | 0.008 | 0.565 | 0.614 | 0.365 | 0.391 | 0.241 | 0.671 | 0.67 | 0.503 | 0.468 |
| *G_iX_* | 0.556 | 0.814 | 0.879 | 0.696 | 0.417 | 0.097 | 0.873 | 0.795 | 0.575 | 0.532 | 0.427 | 0.862 | 0.866 | 0.591 | 0.587 |
| *G_iX/Pi_* | 0.509 | 0.778 | 0.867 | 0.695 | 0.341 | 0.054 | 0.798 | 0.738 | 0.517 | 0.523 | 0.338 | 0.790 | 0.812 | 0.530 | 0.526 |
| *G_iIRT_* | 0.556 | 0.814 | 0.879 | 0.696 | 0.417 | 0.097 | 0.873 | 0.795 | 0.575 | 0.532 | 0.427 | 0.862 | 0.866 | 0.591 | 0.587 |
| *G_iML_* | 0.464 | 0.744 | 0.856 | 0.659 | 0.409 | -0.014 | 0.907 | 0.826 | 0.478 | 0.495 | 0.379 | 0.882 | 0.865 | 0.595 | 0.611 |
| *G_iPC_* | 0.482 | 0.789 | 0.867 | 0.683 | 0.402 | 0 | 0.876 | 0.805 | 0.502 | 0.5 | 0.4 | 0.856 | 0.849 | 0.58 | 0.585 |
| *RPC_iX_* | 0.459 | 0.743 | 0.870 | 0.684 | 0.379 | 0.083 | 0.823 | 0.764 | 0.541 | 0.532 | 0.427 | 0.860 | 0.784 | 0.593 | 0.584 |
| *RPC_iX/Pi_* | 0.449 | 0.733 | 0.874 | 0.698 | 0.336 | 0.040 | 0.773 | 0.733 | 0.506 | 0.567 | 0.357 | 0.817 | 0.764 | 0.522 | 0.541 |
| *RPC_iIRT_* | 0.459 | 0.743 | 0.870 | 0.684 | 0.379 | 0.083 | 0.823 | 0.764 | 0.541 | 0.532 | 0.427 | 0.860 | 0.784 | 0.593 | 0.584 |
| *RPC_iML_* | 0.423 | 0.710 | 0.871 | 0.650 | 0.355 | 0.000 | 0.897 | 0.833 | 0.454 | 0.534 | 0.404 | 0.897 | 0.802 | 0.630 | 0.659 |
| *RPC_iPC_* | 0.425 | 0.764 | 0.879 | 0.672 | 0.36 | 6E-08 | 0.868 | 0.809 | 0.469 | 0.529 | 0.421 | 0.875 | 0.788 | 0.621 | 0.631 |

|  | V16 | V17 | V18 | V19 | V20 | V21 | V22 | V23 | V24 | V25 | V26 | V27 | V28 | V29 | V30 | SUM |
| --- | --- | --- | --- | --- | --- | --- | --- | --- | --- | --- | --- | --- | --- | --- | --- | --- |
| *p* | 0,612 | 0,735 | 0,857 | 0,673 | 0,673 | 0,633 | 0,653 | 0,612 | 0,714 | 0,571 | 0,673 | 0,469 | 0,510 | 0,367 | 0,245 |  |
| $\sigma_{i}^{2}=p\left(1-p\right)$ | 0,237 | 0,195 | 0,122 | 0,220 | 0,220 | 0,232 | 0,227 | 0,237 | 0,204 | 0,245 | 0,220 | 0,249 | 0,250 | 0,232 | 0,185 | 5.574 |
| *B* (IRT) | 0,598 | -0,110 | -1,017 | 0,256 | 0,256 | 0,486 | 0,372 | 0,598 | 0,016 | 0,818 | 0,256 | 1,360 | 1,143 | 1,914 | 2,641 |  |
| $\lambda_{i\text{θ}ML}$ | 0,298 | 0,246 | 0,439 | 0,678 | 0,435 | 0,528 | 0,562 | 0,480 | 0,398 | 0,507 | 0,363 | 0,097 | 0,413 | 0,364 | 0,399 |  |
| $\lambda_{i\text{θPC}}$ | 0,342 | 0,263 | 0,473 | 0,666 | 0,455 | 0,585 | 0,600 | 0,535 | 0,446 | 0,529 | 0,393 | 0,131 | 0,444 | 0,429 | 0,455 |  |
| Somers D*_iX_* | 0,442 | 0,355 | 0,694 | 0,731 | 0,519 | 0,706 | 0,717 | 0,689 | 0,608 | 0,638 | 0,521 | 0,309 | 0,477 | 0,595 | 0,655 |  |
| *σ_i_* ×*R_iX_* | 0,188 | 0,126 | 0,153 | 0,289 | 0,201 | 0,290 | 0,290 | 0,276 | 0,211 | 0,255 | 0,198 | 0,104 | 0,217 | 0,226 | 0,202 | 5.780 |
| *σ_i_* ×*RPC_iX_* | 0,228 | 0,163 | 0,237 | 0,362 | 0,272 | 0,357 | 0,359 | 0,319 | 0,267 | 0,317 | 0,266 | 0,162 | 0,248 | 0,308 | 0,287 | 7.707 |
| *σ_i_* ×*G_iX_* | 0,227 | 0,166 | 0,249 | 0,359 | 0,257 | 0,350 | 0,353 | 0,344 | 0,286 | 0,329 | 0,253 | 0,161 | 0,252 | 0,297 | 0,297 | 7.894 |
| *σ_i_* ×*D_iX_* | 0,215 | 0,157 | 0,243 | 0,343 | 0,243 | 0,340 | 0,341 | 0,336 | 0,275 | 0,316 | 0,244 | 0,154 | 0,238 | 0,287 | 0,282 | 7.588 |
| *R_iX_* | 0,385 | 0,286 | 0,438 | 0,617 | 0,429 | 0,601 | 0,609 | 0,566 | 0,468 | 0,516 | 0,422 | 0,209 | 0,434 | 0,468 | 0,470 |  |
| *R_iX/Pi_* | 0,397 | 0,289 | 0,442 | 0,558 | 0,411 | 0,613 | 0,614 | 0,585 | 0,482 | 0,494 | 0,416 | 0,252 | 0,422 | 0,547 | 0,578 |  |
| *R_iIRT_* | 0,371 | 0,276 | 0,411 | 0,585 | 0,433 | 0,602 | 0,601 | 0,571 | 0,478 | 0,526 | 0,425 | 0,292 | 0,399 | 0,521 | 0,500 |  |
| *R_iML_* | 0.315 | 0.261 | 0.464 | 0.717 | 0.460 | 0.558 | 0.595 | 0.508 | 0.421 | 0.536 | 0.383 | 0.103 | 0.436 | 0.384 | 0.422 |  |
| *R_iPC_* | 0.342 | 0.263 | 0.473 | 0.666 | 0.455 | 0.585 | 0.6 | 0.535 | 0.446 | 0.529 | 0.393 | 0.131 | 0.444 | 0.429 | 0.455 |  |
| *G_iX_* | 0.465 | 0.376 | 0.713 | 0.766 | 0.548 | 0.727 | 0.741 | 0.706 | 0.634 | 0.664 | 0.540 | 0.322 | 0.504 | 0.617 | 0.691 |  |
| *G_iX/Pi_* | 0.453 | 0.380 | 0.721 | 0.659 | 0.477 | 0.735 | 0.743 | 0.705 | 0.629 | 0.585 | 0.515 | 0.311 | 0.500 | 0.649 | 0.797 |  |
| *G_iIRT_* | 0.465 | 0.376 | 0.713 | 0.766 | 0.548 | 0.727 | 0.741 | 0.706 | 0.634 | 0.664 | 0.540 | 0.322 | 0.504 | 0.617 | 0.691 |  |
| *G_iML_* | 0.365 | 0.329 | 0.707 | 0.837 | 0.568 | 0.688 | 0.728 | 0.649 | 0.563 | 0.670 | 0.492 | 0.214 | 0.460 | 0.541 | 0.649 |  |
| *G_iPC_* | 0.404 | 0.333 | 0.714 | 0.78 | 0.549 | 0.706 | 0.728 | 0.674 | 0.6 | 0.653 | 0.511 | 0.237 | 0.47 | 0.573 | 0.685 |  |
| *RPC_iX_* | 0.467 | 0.370 | 0.676 | 0.771 | 0.580 | 0.740 | 0.754 | 0.655 | 0.592 | 0.640 | 0.568 | 0.324 | 0.497 | 0.639 | 0.668 |  |
| *RPC_iX/Pi_* | 0.433 | 0.408 | 0.697 | 0.690 | 0.519 | 0.765 | 0.778 | 0.668 | 0.604 | 0.594 | 0.563 | 0.322 | 0.495 | 0.685 | 0.822 |  |
| *RPC_iIRT_* | 0.467 | 0.370 | 0.676 | 0.771 | 0.580 | 0.740 | 0.754 | 0.655 | 0.592 | 0.640 | 0.568 | 0.324 | 0.497 | 0.639 | 0.668 |  |
| *RPC_iML_* | 0.344 | 0.348 | 0.687 | 0.868 | 0.615 | 0.714 | 0.751 | 0.622 | 0.543 | 0.688 | 0.547 | 0.224 | 0.470 | 0.566 | 0.680 |  |
| *RPC_iPC_* | 0.389 | 0.355 | 0.706 | 0.816 | 0.609 | 0.714 | 0.738 | 0.627 | 0.581 | 0.654 | 0.572 | 0.247 | 0.478 | 0.605 | 0.712 |  |

**Calculation of the scores**

Calculation of the raw score is straightforward as well as is the non-linear score based on difficulty level of the items $\text{θ}_{NonL}=\text{θ}_{PI}=\sum_{i=1}^{k} g_{i}/p_{i}$. Principal component scores, factor scores, and theta scores from the one-parameter logistic (Rasch) model can be obtained as follows.

# *Principal component analysis*

In IBM SPSS, the syntax for principal components is FACTOR /VARIABLES V1 to V30 /MISSING LISTWISE /ANALYSIS V1 to V30 /PRINT INITIAL EXTRACTION /CRITERIA FACTORS(1) ITERATE(25) /EXTRACTION PC /ROTATION NOROTATE /SAVE REG(ALL) /METHOD=CORRELATION. Alternatively, in SAS, the PROC FACTOR statement with the specification METHOD=PRINCIPAL can be used. With R, the **principal( )** function in the [psych](http://cran.r-project.org/web/packages/psych/index.html) package can be used (see e.g., <https://cran.r-project.org/web/packages/psychTools/vignettes/overview.pdf> or <https://www.statmethods.net/advstats/factor.html>)

# *Factor analysis*

In IBM SPSS, the syntax for Factor analysis with maximum likelihood estimates is FACTOR /VARIABLES V1 to V30 /MISSING LISTWISE /ANALYSIS V1 to V30 /PRINT INITIAL EXTRACTION /CRITERIA FACTORS(1) ITERATE(25) /EXTRACTION ML /ROTATION NOROTATE /SAVE REG(ALL) /METHOD=CORRELATION. Alternatively, in SAS, the PROC FACTOR statement with the specification METHOD=ML can be used. With R, the **factor.pa( ) function in the** [psych](http://cran.r-project.org/web/packages/psych/index.html)package can be used (see e.g., <https://cran.r-project.org/web/packages/psychTools/vignettes/overview.pdf> or <https://www.statmethods.net/advstats/factor.html>).

# *Rasch- and IRT parameters*

For Rasch and IRT parameters (the item parameter B and person parameter Theta), usually, specific software packages are used. In the article, the software package OPML (Verhelst, N. D., Glas, C. A. W. & Verstralen, H. H. F. M. (1995). *One Parametric Logistic Model OPLM.* Arnhem: CITO) was in use. IBM SPSS do not support IRT analysis. In SAS, PROC IRT provides for unidimensional analysis (see further <https://support.sas.com/rnd/app/stat/procedures/irt.html>). With R, the parameters can be obtained, for instance, by the packages ltm (<https://cran.r-project.org/web/packages/ltm/ltm.pdf>), equateIRT (<https://cran.r-project.org/web/packages/equateIRT/index.html>) or irt (<https://cran.r-project.org/web/packages/irt/irt.pdf>).

**Calculation of the estimates of weight factors**

In the article, such estimators were discussed as product-moment correlation coefficient (PMC; Pearson, 1896), polychoric correlation coefficient (*RPC*; Pearson, 1900, 1913), Goodman–Kruskal gamma (*G*; Goodman & Kruskal, 1954), Somers delta (*D*; Somers, 1962), dimension-corrected *G* (*G*_2_; Metsämuuronen, 2021a), and dimension-corrected *D* (*D*_2_; Metsämuuronen, 2020b, 2021a). Although all of these were not used in the empirical section, options for their calculation are given here.

*PMC*

In IBM SPSS, the syntax for PMC is CORRELATIONS /VARIABLES=item score MISSING=PAIRWISE. In SAS, the command PROC CORR provides PMC. In R, PMC can be calculated by cor(x, y, method = c("pearson")) (see http://www.sthda.com/english/wiki/correlation-test-between-two-variables-in-r).

*RPC*

In IBM SPSS, the syntax for *RPC* is not available although some macros are (e.g., Lorenzo-Seva, U., & Ferrando, P.J. (2015). POLYMAT-C: a comprehensive SPSS program for computing the polychoric correlation matrix. *Behavior Research Methods, 47***,** 884–889. <https://doi.org/10.3758/s13428-014-0511-x>). In SAS, the command PROC CORR provides *RPC*. With R, *R_PC_* can be calculated by [*CorPolychor*](https://rdrr.io/cran/DescTools/man/CorPolychor.html)*(x, y, ML = FALSE, control =* list*(), std.err = FALSE, maxcor=.9999)## S3 method for class 'CorPolychor'* print*(x, digits =* max*(3,* getOption*("digits") - 3), ...)* (see, <https://rdrr.io/cran/DescTools/man/CorPolychor.html>). In the article, RPCs between items and a score variables were calculated manually by using Zaionts procedure of Martinson & Hamdan (1972) two-step estimator (Zaionts, C. (2021). *Real Statics Using Excel.* Polychoric Correlation using Solver. <http://www.real-statistics.com/correlation/polychoric-correlation/polychoric-correlation-using-solver/>).

*G & G*_2_

In IBM SPSS, the syntax for *G* is CROSSTABS /TABLES=item BY Score /STATISTICS=GAMMA. In SAS, the command PROC FREQ provides *G* by specifying the TEST statement by GAMMA, SMDCR options. With R, *G* is calculated by [*GoodmanKruskalGamma*](https://rdrr.io/cran/DescTools/man/GoodmanKruskalGamma.html)*(x, y = NULL, conf.level = NA, ...)* (see, <https://rdrr.io/cran/DescTools/man/>). The values of *G*_2_ are identical with *G* in the binary settings. However, as an example of calculating *G*_2_, if the item had three categories in the scale of more, *G*_2_ can be calculated by knowing the observed value of *G* and number of categories in the item (see Metsämuuronen, J. (2021a). Goodman–Kruskal gamma and dimension-corrected gamma in educational measurement settings. *International Journal of Educational Methodology, 7*(1), 95–118. <https://doi.org/10.12973/ijem.7.1.95>): $G_{2}=G\times\left(1+\left(1-abs\left(G\right)\right)\times A\right)$, $A=\left(df\left(g\right)-1\right)/df\left(g\right)\times\left(1-1/df\left(g\right)\right)^{2}$, and *df*(*g*) = (number of categories in the item – 1). As an example, assuming *G* = 0.50 and the item scale with three categories (e.g., 0, 1, and 2), *df*(*g*) = 3 – 1 = 2, $A=0.5\times0.25=0.125$, and $G_{2}=0.5\times\left(1+\left(1-0.5\right)\times0.125\right)=0.531$.

*D & D*_2_

In IBM SPSS, the syntax for *D* is CROSSTABS /TABLES=item BY Score /STATISTICS=D. In SAS, the command PROC FREQ provides *D* by specifying the TEST statement by D, SMDCR options. with R, *D* can be calculated by [*SomersDelta*](https://rdrr.io/cran/DescTools/man/SomersDelta.html)*(x, y = NULL, direction =* c*("row", "column"), conf.level = NA, ...)* (see, <https://rdrr.io/cran/DescTools/man/>). The values of *D*_2_ are identical with *D* in the binary settings. However, as an example of calculating *G*_2_, if the item had three categories in the scale of more, *D*_2_ can be calculated by knowing the observed *D* and *df*(*g*) (see the corrected form in Metsämuuronen, J. (2021a). Goodman–Kruskal gamma and dimension-corrected gamma in educational measurement settings. *International Journal of Educational Methodology, 7*(1), 95–118. <https://doi.org/10.12973/ijem.7.1.95>): $D_{2}=D\times\left(1+\left(1-abs\left(D\right)\right)\times A\right)$, $A=\left(df\left(g\right)-1\right)/df\left(g\right)\times\left(1-1/df\left(g\right)\right)^{2}$, and *df*(*g*) = (number of categories in the item – 1). As an example, assuming *D* = 0.50 and the item scale with three categories (0, 1, and 2), *df*(*g*) = 3 – 1 = 2, $A=0.5\times0.25=0.125$ and$D_{2}=0.5\times\left(1+\left(1-0.5\right)\times0.125\right)=0.531$.

**Calculation of the estimates of reliability**

All the estimators of reliability in the article can be calculated manually by knowing the score variable, item–score correlations, and loadings (see above). Examples of the calculations are given here.

*Alpha and MEC-corrected alpha*

For calculating a traditional and MEC-corrected coefficient alpha for the raw score (*X*), item–score correlations (here, *RPC_iX_* as an example) and related statistics are as follows:

| items | V1 | V2 | V3 | V4 | V5 | **…** | V26 | V27 | V28 | V29 | V30 | SUM |
| --- | --- | --- | --- | --- | --- | --- | --- | --- | --- | --- | --- | --- |
| *p*(1–*p*) = *σ_i_*^2^ | 0.092 | 0.075 | 0.162 | 0.137 | 0.137 | **…** | 0.22 | 0.249 | 0.25 | 0.232 | 0.185 | 5.574 |
| *R*it = *R_iX_* | 0.082 | 0.114 | 0.253 | 0.168 | 0.09 | **…** | 0.422 | 0.209 | 0.434 | 0.468 | 0.47 |  |
| *σ_i_* ×*R_iX_* | 0.272 | 0.416 | 0.627 | 0.454 | 0.244 | **…** | 0.198 | 0.104 | 0.217 | 0.226 | 0.202 | 5.780 |
| *RPC_iX_* | 0.459 | 0.743 | 0.87 | 0.684 | 0.379 | **…** | 0.568 | 0.324 | 0.497 | 0.639 | 0.668 |  |
| *σ_i_* ×*RPC_iX_* | 0.139 | 0.203 | 0.351 | 0.253 | 0.14 | **…** | 0.266 | 0.162 | 0.248 | 0.308 | 0.287 | 7.707 |

By substituting the values in the traditional formula of alpha (Eq. 1), we get an estimate of reliability

$$\rho_{\alpha}=\rho_{\alpha\_RiX}=\frac{k}{k-1}\left(1-\frac{\sum_{i=1}^{k} \sigma_{i}^{2}}{\left(\sum_{i=1}^{k} \sigma_{i}\times\rho_{iX}\right)^{2}}\right)=\frac{30}{29}\left(1-\frac{5.574}{5.780^{2}}\right)=0.862$$

and, if a MEC-corrected estimate of correlation is used, here *RPC* as an example (Eq. 13), we get an estimate by a MEC-corrected alpha

$\rho_{\alpha\_RPCiX}=\frac{k}{k-1}\left(1-\frac{\sum_{i=1}^{k} \sigma_{i}^{2}}{\left(\sum_{i=1}^{k} \sigma_{i}\times RPC_{iX}\right)^{2}}\right)=\frac{30}{29}\left(1-\frac{5.574}{7.707^{2}}\right)=0.937$.

*Theta*

For calculating the traditional coefficient alpha based on the principal component score (*PC*), principal component loading (*λ_PC_*) and related statistics are as follows:

| items | V1 | V2 | V3 | V4 | V5 | **…** | V26 | V27 | V28 | V29 | V30 | SUM |
| --- | --- | --- | --- | --- | --- | --- | --- | --- | --- | --- | --- | --- |
| Principal component loadings $\lambda_{i\text{θ}PC}$ | 0.229 | 0.425 | 0.669 | 0.466 | 0.227 | **…** | 0.393 | 0.131 | 0.444 | 0.429 | 0.455 |  |
| $\lambda_{i\text{θ}PC}^{2}$ | 0.052 | 0.181 | 0.448 | 0.217 | 0.052 | **…** | 0.154 | 0.017 | 0.197 | 0.184 | 0.207 | 6.648 |

By substituting the values in the traditional formula of theta (Eq. 2), we get an estimate of reliability

$\rho_{TH}=\frac{k}{k-1}\left(1-\frac{1}{\sum_{i=1}^{k} \lambda_{i\text{θ}PC}^{2}}\right)=\frac{30}{29}\times\left(1-\frac{1}{6.648}\right)=0.879$.

Although no MEC-corrected theta (such as Eqs. 16 and 17) was calculated in the article, those would be easy to calculate by substituting $\lambda_{i}^{2}$ with a less MEC-affected estimator of items–score correlation.

*Omega*

For calculating the traditional coefficient omega based on the factor score variable (*FA*), maximum likelihood estimates of factor loading ($\lambda_{i\text{θ}ML}$) and related statistics are as follows:

| items | V1 | V2 | V3 | V4 | V5 | **…** | V26 | V27 | V28 | V29 | V30 |  |
| --- | --- | --- | --- | --- | --- | --- | --- | --- | --- | --- | --- | --- |
| factor loadings $\lambda_{i\text{θ}ML}$ | 0.204 | 0.376 | 0.635 | 0.42 | 0.213 | **…** | 0.363 | 0.097 | 0.413 | 0.364 | 0.399 | 12.38 |
| $\lambda_{i\text{θ}ML}^{2}$ | 0.042 | 0.141 | 0.403 | 0.176 | 0.045 | **…** | 0.132 | 0.009 | 0.171 | 0.132 | 0.159 |  |
| 1 – $\lambda_{i\text{θ}ML}^{2}$ | 0.958 | 0.859 | 0.597 | 0.824 | 0.955 | **…** | 0.868 | 0.991 | 0.829 | 0.868 | 0.841 | 24.09 |

By substituting the values in the traditional formula of omega (Eq. 3), we get an estimate of reliability

$\rho_{\omega}=\frac{\left(\sum_{i=1}^{k} \lambda_{i\text{θ}ML}\right)^{2}}{\left(\sum_{i=1}^{k} \lambda_{i\text{θ}ML}\right)^{2}+\sum_{i=1}^{k} \left(1-\lambda_{i\text{θ}ML}^{2}\right)}=\frac{12.375^{2}}{12.375^{2}+24.093}=0.864$.

Although no MEC-corrected omega (such as Eqs. 18 and 19) was calculated in the article, those would be easy to calculate by substituting $\lambda_{i}^{2}$ with a less MEC-affected estimator of item–score correlation.

*Rho and MEC-corrected rho*

For calculating a traditional and MEC-corrected coefficient rho (maximal reliability) for the factor score variable (*FA*), maximum likelihood estimates of factor loading ($\lambda_{i\text{θ}ML}$) and an alternative substitute being less affected by MEC (here, *RPC_i_*_θ_ as an example) and related statistics are as follows:

| items | V1 | V2 | V3 | V4 | V5 | **…** | V26 | V27 | V28 | V29 | V30 | SUM |
| --- | --- | --- | --- | --- | --- | --- | --- | --- | --- | --- | --- | --- |
| factor loadings $\lambda_{i\text{θ}ML}$ | 0.204 | 0.376 | 0.635 | 0.42 | 0.213 | **…** | 0.363 | 0.097 | 0.413 | 0.364 | 0.399 |  |
| $\lambda_{i\text{θ}ML}^{2}$ | 0.042 | 0.141 | 0.403 | 0.176 | 0.045 | **…** | 0.132 | 0.009 | 0.171 | 0.132 | 0.159 |  |
| 1 – $\lambda_{i\text{θ}ML}^{2}$ | 0.958 | 0.859 | 0.597 | 0.824 | 0.955 | **…** | 0.868 | 0.991 | 0.829 | 0.868 | 0.841 |  |
| $\lambda_{i\text{θ}ML}^{2}/\left(1-\lambda_{i\text{θ}ML}^{2}\right)$ | 0.043 | 0.165 | 0.676 | 0.214 | 0.048 | **…** | 0.152 | 0.009 | 0.206 | 0.153 | 0.189 | 8.462 |
| $RPC_{i\text{θ}}$ | 0.423 | 0.71 | 0.871 | 0.65 | 0.355 | **…** | 0.547 | 0.224 | 0.47 | 0.566 | 0.68 |  |
| $RPC_{i\text{θ}}^{2}$ | 0.179 | 0.504 | 0.759 | 0.423 | 0.126 | **…** | 0.299 | 0.05 | 0.221 | 0.32 | 0.462 |  |
| 1 –$RPC_{i\text{θ}}^{2}$ | 0.821 | 0.496 | 0.241 | 0.577 | 0.874 | **…** | 0.701 | 0.95 | 0.779 | 0.68 | 0.538 |  |
| $RPC_{i\text{θ}}^{2}/\left(1-RPC_{i\text{θ}}^{2}\right)$ | 0.218 | 1.017 | 3.144 | 0.733 | 0.145 | **…** | 0.426 | 0.053 | 0.284 | 0.471 | 0.859 | 31.05 |

By substituting the values in the formula of rho (Eq. 4), we get an estimate of reliability as

$\rho_{MAX}=\rho_{MAX\_\lambda i\text{θ}}=\frac{1}{1+\frac{1}{\sum_{i=1}^{k} \left(\lambda_{i\text{θ}ML}^{2}/\left(1-\lambda_{i\text{θ}ML}^{2}\right)\right)}}=\frac{1}{1+\frac{1}{8.462}}=0.894$,

and, if a MEC-corrected estimate of correlation is used (here, *RPC* as an example), we get an estimate by a MEC-corrected rho as

$\rho_{MAX\_RPCi\text{θ}}=\frac{1}{1+\frac{1}{\sum_{i=1}^{k} \left(RPC_{i\text{θ}}^{2}/\left(1-RPC_{i\text{θ}}^{2}\right)\right)}}=\frac{1}{1+\frac{1}{31.054}}=0.969$.
